# Kinesin-1 promotes chondrocyte maintenance during skeletal morphogenesis

**DOI:** 10.1371/journal.pgen.1006918

**Published:** 2017-07-17

**Authors:** Adrian Santos-Ledo, Marina Garcia-Macia, Philip D. Campbell, Marta Gronska, Florence L. Marlow

**Affiliations:** 1 Department of Developmental and Molecular Biology. Albert Einstein College of Medicine, Bronx, New York, United States of America; 2 Institute of Genetic Medicine. Newcastle University, Newcastle Upon Tyne, United Kingdom; 3 Institute for Cellular and Molecular Biosciences. Newcastle University, Newcastle Upon Tyne, United Kingdom; 4 Institute of Cellular Medicine. Newcastle University, Newcastle Upon Tyne, United Kingdom; 5 Department of Neuroscience. Albert Einstein College of Medicine, Bronx, New York, United States of America; 6 Cell Developmental and Regenerative Biology Department. Icahn School of Medicine at Mount Sinai. New York, New York, United States of America; Stowers Institute, UNITED STATES

## Abstract

During skeletal morphogenesis diverse mechanisms are used to support bone formation. This can be seen in the bones that require a cartilage template for their development. In mammals the cartilage template is removed, but in zebrafish the cartilage template persists and the bone mineralizes around the cartilage scaffold. Remodeling of unmineralized cartilage occurs via planar cell polarity (PCP) mediated cell rearrangements that contribute to lengthening of elements; however, the mechanisms that maintain the chondrocyte template that supports perichondral ossification remain unclear. We report double mutants disrupting two zebrafish *kinesin-I* genes (hereafter *kif5Blof*) that we generated using CRISPR/Cas9 mutagenesis. We show that zygotic Kif5Bs have a conserved function in maintaining muscle integrity, and are required for cartilage remodeling and maintenance during craniofacial morphogenesis by a PCP-distinct mechanism. Further, *kif5Blof* does not activate ER stress response genes, but instead disrupts lysosomal function, matrix secretion, and causes deregulated autophagic markers and eventual chondrocyte apoptosis. Ultrastructural and transplantation analysis reveal neighboring cells engulfing extruded *kif5Blof* chondrocytes. Initial cartilage specification is intact; however, during remodeling, *kif5Blof* chondrocytes die and the cartilage matrix devoid of hypertrophic chondrocytes remains and impedes normal ossification. Chimeric and mosaic analyses indicate that Kif5B functions cell-autonomously in secretion, nuclear position, cell elongation and maintenance of hypertrophic chondrocytes. Interestingly, large groups of wild-type cells can support elongation of neighboring mutant cells. Finally, mosaic expression of *kif5Ba*, but not *kif5Aa* in cartilage rescues the chondrocyte phenotype, further supporting a specific requirement for Kif5B. Cumulatively, we show essential Kif5B functions in promoting cartilage remodeling and chondrocyte maintenance during zebrafish craniofacial morphogenesis.

## Author summary

During skeletal morphogenesis diverse mechanisms are used to support bone formation, for example some bones require a cartilage template. In mammals the cartilage template is removed, but in zebrafish the cartilage template persists and the bone mineralizes around the cartilage scaffold. Remodeling of unmineralized cartilage occurs via planar cell polarity (PCP) mediated cell rearrangements that contribute to lengthening of elements. We identified a conserved role for the Kinesin-1 heavy chain, *kif5B*, in maintaining muscle integrity and a novel role in cartilage development during craniofacial morphogenesis. In chondrogenesis Kif5B acts by a distinct mechanism from PCP signaling and does not activate the ER stress response genes, but instead involves regulation of lysosomal function, matrix secretion, and autophagy. When *kif5B* is lost, autophagic markers are deregulated leading to eventual chondrocyte apoptosis. Chimeric and mosaic analyses indicate that Kif5B functions cell-autonomously in secretion, nuclear position, cell elongation and maintenance. Interestingly, large groups of wild-type cells, likely via their matrix, support elongation of neighboring mutant cells. Cumulatively, our study reveals Kif5B’s essential role in promoting cartilage remodeling and chondrocyte maintenance during craniofacial morphogenesis.

## Introduction

Intramembranous ossification–formation of bone within connective tissue, and endochondral or perichondral ossification–formation of bone via mineralization and reabsorption of cartilage anlage (endochondral) or mineralization around a persisting cartilage scaffold (perichondral) that serve as templates for later bone formation, and apoptotic remodeling of unmineralized cartilage are mechanisms that contribute to skeletal tissue morphogenesis [1]. The zebrafish jaw is a genetically tractable system to investigate the molecular and genetic basis of cartilage and bone morphogenesis [2]. During morphogenesis the chondrocytes of long bones, endochondral or perichondral bones in teleost fish, including those in the jaw, elongate and orient their primary cilia and microtubule organizing centers (MTOC), and, via oriented intercalation, the discoid cells align in a stacked configuration [3]. Disruption of Wnt Planar Cell Polarity (PCP) genes *pipetail* (*wnt5b* [4]) and *knypek* (*gpc4 [5]*) or Fat/Dachsous depletion [3] disrupts cartilage cell polarity and stacking. Mutations in vesicular trafficking machinery components impair cellular secretion and extracellular matrix (ECM) deposition [6, 7], in some cases lead to ER stress response activation [8, 9] and disrupt chondrogenesis [8–10]. Although unmineralized cartilage remodels via conserved PCP-mediated polarization and cell rearrangements, the mechanisms that maintain the chondrocyte template that supports perichondral ossification remain unclear.

Apoptosis, or programmed cell death (PCD), a genetically regulated form of cell death that eliminates damaged or infected cells, is vital for normal development [11]. The cellular and molecular mechanisms underlying PCD can be cell-type specific. For example, molecularly distinct mechanisms of apoptosis have been described in neurons [12] and chondrocytes [13]. The uniquely inaccessible microenvironments of these cells limit phagocyte access and render them susceptible to damage caused by cellular detritus associated with typical apoptosis. Cellular hallmarks of chondrocyte cell death have been described in mammalian systems *in vivo*, including expansion of rough endoplasmic reticulum (rER) and Golgi, pronounced autophagic vacuoles, and patchy chromatin condensations [13]. Apoptosis with autophagic characteristics also occurs in other systems with important secretory functions [14]. Macroautophagy (referred to as autophagy hereafter) is the cellular process responsible for catabolizing organelles and cellular components via autophagosomes and lysosomes during stress conditions [15]. Autophagy has been implicated in a myriad of developmental and differentiation processes [16], including autophagic cell death during embryogenesis [17]. Aberrant autophagy has been linked to bone and cartilage diseases such as osteoarthritis [18]. Cell death associated with disease may result from impaired chondrocyte function in ECM maintenance or remodeling [19, 20]. However, the molecular mechanisms underlying chondrocyte maintenance and cell death remain poorly understood because chondrocyte death is rarely observed in healthy tissue *in situ*, and when observed in culture does not resemble the histological and molecular features described *in vivo* [13].

Kinesin motors transport or tether components to specific subcellular locations. The vertebrate Kinesin-1 family (also called Kif5s) includes *kif5A*, *B*, and *C* genes [21]. *kif5A* and *kif5C* are expressed exclusively in the nervous system and when mutated cause human diseases [22, 23]. *Kif5B* is broadly expressed but its *in vivo* functions are largely unknown. *kif5B* mutant mice are embryonic lethal [24] and muscle-specific *kif5B* knock-out disrupts skeletal muscle differentiation [25]. Maternally provided zebrafish Kif5Ba regulates germ cell specification and embryonic patterning [26]. In *in vitro* contexts, Kif5B has been linked to organelle transport [24, 27]. However, whether *kif5B* contributes to skeletal morphogenesis remains unknown.

Here we report a novel role for Kif5B in promoting chondrocyte maintenance. We generated *kif5Ba* and *kif5Bb* compound mutants; hereafter, referred to as Kif5B loss-of-function (*kif5Blof*), and uncovered an essential Kif5B function in craniofacial development. Genetic interactions with Wnt PCP mutants indicate distinct roles for PCP and *kif5B* in cartilage morphogenesis. Initial cartilage specification is unaffected as early chondrocytic markers are intact. Later, when remodeling should occur, several autophagic components, including the inhibitor Tor (Target of Rapamycin), are disrupted in *kif5Blof* chondrocytes, which consequently undergo cell death. Along with cellular features characteristic of those described for chondrocyte apoptosis *in vivo*, neighboring cells engulfed and extruded *kif5Blof* chondrocytes from the cartilage leaving behind a matrix scaffold largely devoid of hypertrophic chondrocytes. Chimeric analyses indicate a cell-autonomous requirement for Kif5B in secretion, nuclear position, cell elongation and maintenance. Interestingly, large groups of transplanted wild-type (Wt) cells supported elongation of neighboring mutant cells, revealing nonautonomous effects. Finally, mosaically expressed *kif5Ba*, but not *kif5Aa*, in cartilage rescues the chondrocyte phenotypes of *kif5Blof*, further supporting a specific requirement for Kif5B. Taken together, our study supports a role for Kif5B in cartilage remodeling and chondrocyte maintenance during craniofacial development.

## Results

### *kif5Bs* redundantly contribute to craniofacial development

**Fig 1:**
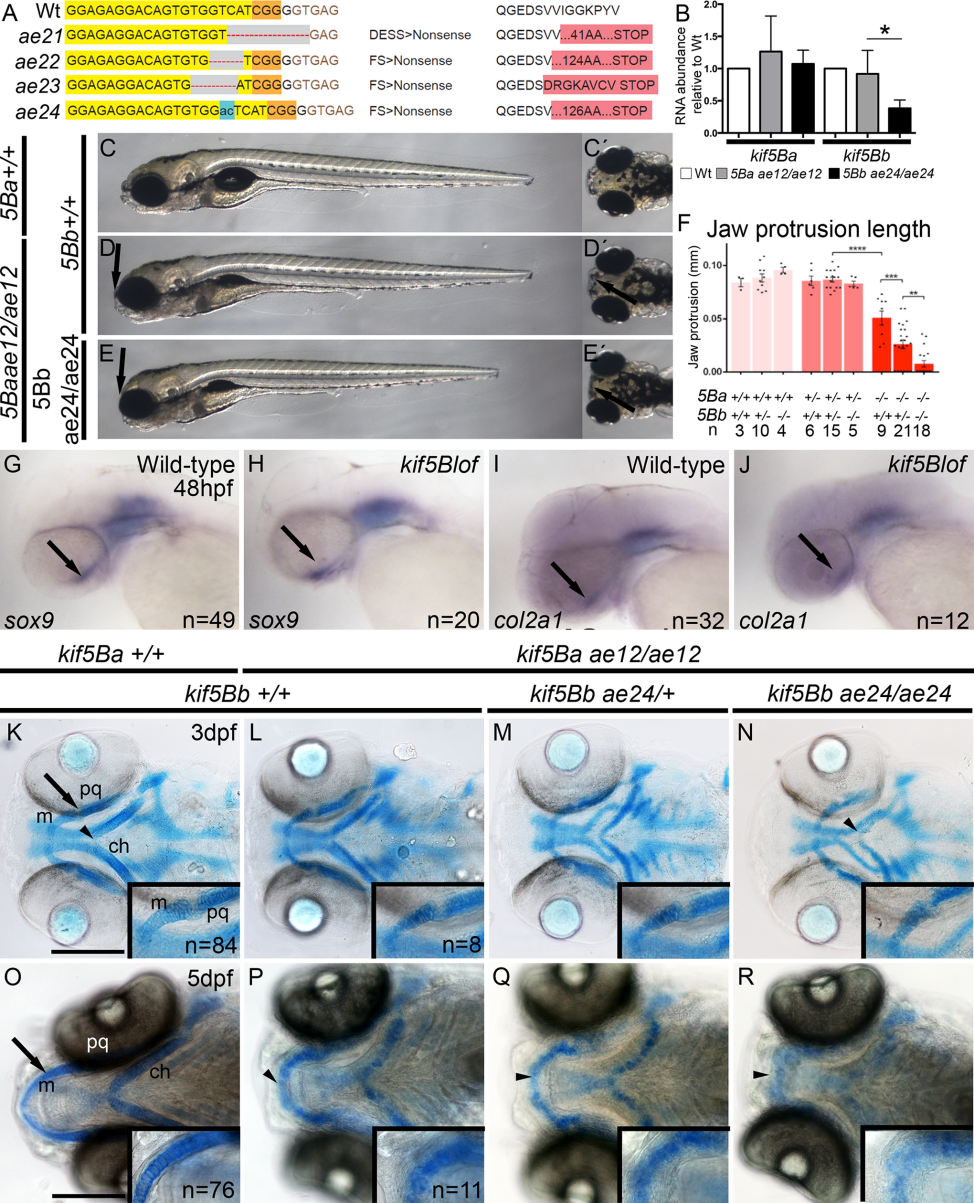
*kif5Bs* contribute to craniofacial development. A) Using Crispr-CAS9 mutagenesis to target the N-terminus, *ae21* (9bp deletion), *ae22* (4bp deletion), *ae23* (5bp deletion) and *ae24* (2bp insertion causing a frame shift and early stop codon) *kif5Bb* mutant alleles were generated. Exon is indicated in yellow, PAM region in orange, red dash or blue areas indicate mutated regions. B) qRT-PCR for *kif5Ba* and *kif5Bb* in *kif5Ba*^*ae12/ae12*^ and *kif5Bb*^*ae24/ae24*^ embryos. *kif5Bb* transcripts undergo NMD in *kif5Bb*^*ae24/ae24*^ mutants. One-way ANOVA * p<0.05. C-E) Live images of 6dpf *kif5Bs* mutants. Mutation of *kif5Ba* causes incompletely penetrant jaw protrusion defects (arrow in D and D´). *kif5Ba*^*ae12/ae12*^*Kif5Bb*^*ae24/ae24*^ mutants exhibit a fully penetrant phenotype, including flat heads (arrows in E and E´). F) Measurements of the jaw protrusion in each genotype. One-way ANOVA ** p<0.01, *** p<0.001, **** p<0.0001. G-J) Whole-mount *in situ* hybridization of *sox9a* (G, H) and *col2a1* (I, J) revealed no differences between Wt and mutants. Arrows indicate the cartilage region. K-N) Alcian blue staining at 3 dpf, arrow indicates inset region. Intact cartilage elements in *kif5blof* are shorter and mediolaterally broader, the angle at the ceratohyal cartilage intersection is wider (arrowheads in K, N). Alcian blue stain reveals defective chondrocyte stacking (insets in K-N). O-R) Alcian blue staining at 5 dpf, arrow indicates the inset region. Meckel´s cartilage has extended anteriorly (O) in Wt but not in *kif5Blof* mutants. (P-R) the Meckel´s and ceratohyal cartilages are curved in *kif5Blof* (arrowheads in O-R), Alcian blue staining is diffuse, and cell stacking is absent (insets in P-R). ch: ceratohyal; m: Meckel´s; pq: palatoquadrate. Scale bars: A-H: 500μm; I-P: 50μm.

Similar expression patterns and the comparably mild *kif5Ba* zygotic mutant phenotypes [26, 28] indicated potential redundant zygotic functions of the duplicated zebrafish *kif5B* genes, *kif5Ba* and *kif5Bb*. Therefore, we used CRISPR/Cas9 mutagenesis [29, 30] to generate *kif5Bb* mutant alleles disrupting the N-terminal motor domain (Fig 1A). We recovered and sequenced four distinct alleles, three deletion alleles and a 2 base pair insertion. All alleles were predicted to generate premature stop codons, failed to complement one another, and are likely null alleles (Fig 1A). qRT-PCR revealed nonsense mediated decay of *kif5Bb*^*ae24*^ transcripts, further supporting that this allele is likely null (Fig 1B).

In contrast to *kif5Ba* mutants, which show mild, incompletely penetrant craniofacial defects and maternal-effect phenotypes [26], *kif5Bb* mutants were viable fertile adults. To investigate potential *kif5B* redundancy we examined *kif5Ba* and *kif5Bb* compound mutants (*kif5Ba*^*ae12/ae12*^*Kif5Bb*^*ae24/+*^ and *kif5Ba*^*ae12/ae12*^*Kif5Bb*^*ae24/ae24*^ hereafter called *kif5Blof*). Like muscle specific knock-out mice, which show severe defects in skeletal muscle including defects in myofibril and myotendinous junction assembly [25], muscle structure both in trunk (S1A–S1D Fig) and tail regions (S1E–S1H Fig) was disrupted in *kif5Blof* mutants. Deficits in mouse and zebrafish mutants were also similar at the ultrastructural level, with the M-line of the sarcomeres, initially evident at 3dpf becoming less apparent by 5dpf in zebrafish *kif5Blof* mutants (S1I–S1L Fig). Therefore, *kif5B* appears to have a conserved role in muscle development.

Although other tissues examined, including the central nervous system were comparable to Wt (S2 Fig), prominent jaw defects were apparent in *kif5Blof* mutants. Compared to 5 days post fertilization (dpf) Wt larvae (Fig 1C), a subset of *kif5Ba*^*ae12*^ single mutants had flattened heads and reduced jaw protrusion (Fig 1D and 1F). Zygotic *kif5Ba*^*ae12*^ single mutant phenotypes were enhanced and fully penetrant when one copy of *kif5Bb* was mutant. Jaw protrusion was further impaired in *kif5B* double mutants (Fig 1E and 1F). Unlike *kif5Ba* or *kif5Bb* single mutants, *kif5Blof* (both *kif5Ba*^*ae12/ae12*^*Kif5Bb*^*ae24/+*^ and *kif5Ba*^*ae12/ae12*^*Kif5Bb*^*ae24/ae24*^) were lethal, suggesting that *kif5Ba* and *kif5Bb* have redundant essential zygotic functions.

### Chondrocyte function requires *kif5B*

To determine if cartilage specification was impaired in *kif5B* mtuants we examined chondrocyte markers at 48 hpf (*sox9a* and *col2a1*) [3, 7] and found no differences between Wt and *kif5B* mutants based on *in situ* hybridization (Fig 1G–1J) or qRT-PCR analysis using previously published primers [31] (S3 Fig). At 3 dpf, when no overt morphological phenotypes were apparent, Alcian blue staining (Fig 1O–1R) revealed abnormal chondrogenesis in *kif5Blof*. Although each cartilaginous element was present, they were shorter in *kif5B* mutants (Fig 1L–1N, ceratohyal length is quantified in S4B Fig). At this stage, compared to Wt (Fig 1K), the ceratohyal cartilage angle was less acute in *kif5B* mutants (Fig 1L–1N, S4A Fig). Also the distance between Meckel´s and ceratohyal cartilages and the extension of the latter along the antero-posterior axis was reduced (S4C and S4D Fig), suggesting compaction of the head. Furthermore, at this stage chondroctyes of Wt were stacked with apparent discoid morphology (Fig 1K, inset), but *kif5B* mutant chondrocyte morphology appeared blurry and was difficult to discern, revealing a potential secretion deficit in the acidic polysaccharides that Alcian blue stains (Fig 1L–1N, inset). At 5 dpf when Meckel’s cartilage protrudes beyond the eyes of Wt (Fig 1O), *kif5B* mutant phenotypes were more pronounced (Fig 1P–1R), with faint Alcian blue staining making individual chondrocytes difficult to discern.

**Fig 2:**
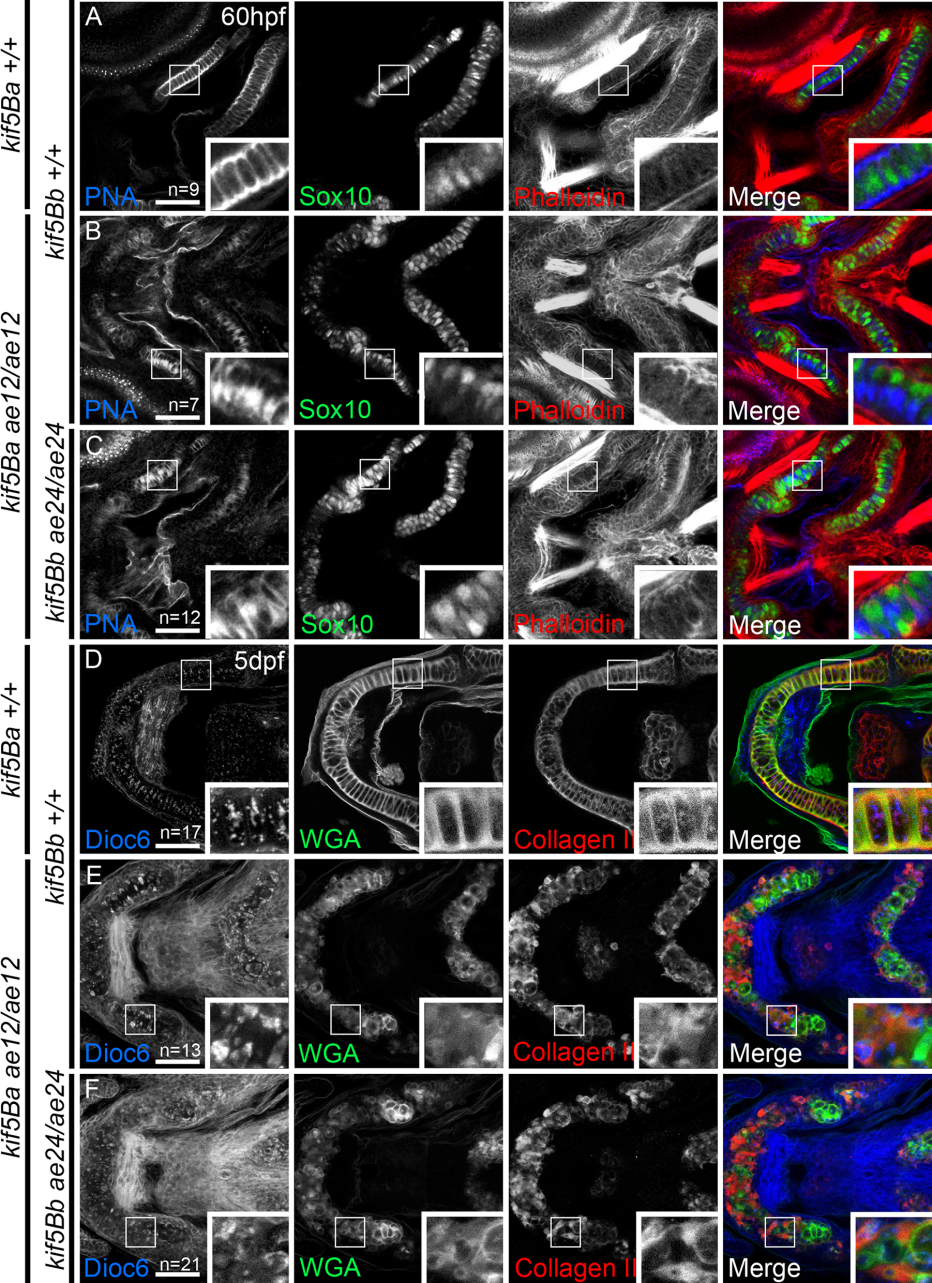
Loss of *kif5B* impairs chondrocyte secretion. A-C) confocal images of Wt (A), single *kif5Ba*^*ae12/ae12*^ mutants (B) and *kif5Ba*^*ae12/ae12*^*Kif5Bb*^*ae24/ae24*^ (C) at 60 hpf. Chondrocytes (Sox10:GFP positive cells) secrete proteins positive for peanut agglutinin (PNA, blue) homogeneously. Secretion in single (B) and compound (C) mutants is deficient. Stacking defects are evident in *kif5Ba*^*ae12/ae12*^*Kif5Bb*^*ae24/ae24*^ (C). D-F) Confocal images of Wt (D), single *kif5Ba*^*ae12/ae12*^ mutants (E) and *kif5Ba*^*ae12/ae12*^*Kif5Bb*^*ae24/ae24*^ (F) at 5 dpf. In Wt (D) Di0C6 (blue) forms variably-sized cellular aggregates. Wheat Germ agglutinin (WGA, green) labels secreted proteins and II-II6B3 marks Collagen II (red) an abundant protein in cartilage. Both are homogeneously secreted in Wt and overlap within ECM (insets in D), although some overlap occurs between Collagen II and Di0C6 (insets in D). In single (E) and compound (F) mutants, Di0C6 forms large patches. Secretion is polarized with little colocalization within ECM (insets in E and F), large patches of Di0C6 colocalize with Collagen II, and cell stacking is perturbed (E, F). Scale bar: 50μm.

The diffuse Alcian blue staining of *kif5B* mtuant chondrocytes was suggestive of impaired secretion; therefore, we examined peanut agglutinin (PNA), which recognizes Gal-β(1,3)-*N*-Acetylgalactosamine, a terminal carbohydrate moiety on proteins, and is commonly used to label mesenchymal condensations [8]. At 60 hours postfertilization (hpf), PNA labels the uniform extracellular space between stacked Wt chondrocytes marked with *sox10*:*GFP* (Fig 2A). In mutants, PNA was detected within chondrocytes (Fig 2B and 2C) and, when outside, was distributed to one side instead of uniform. Moreover, in mutants, the Sox10 reporter occupied one side and prominent PNA marked the other, providing evidence for impaired secretion (Fig 2B and 2C).

To further assess secretion in *kif5B* mutants, we examined WGA (wheat germ agglutinin), which binds to N-acetylglucosamine and N-acetylneuraminic acid (sialic acid) residues of membrane and matrix glycoproteins [10], Di0C6 which labels ER and mitochondria, and collagen II using II-II6B3 antibody. In Wt, Di0C6 puncta were cytoplasmic and variably sized (Fig 2D), WGA was extracellular and delineated cell shapes, and collagen II was primarily extracellular, but also partially overlapped with cytoplasmic Di0C6. Unlike the Di0C6 puncta observed in Wt, large Di0C6 aggregates were apparent in *kif5Ba* single or *kif5B* double mutants (Fig 2E and 2F), indicating abnormal ER and mitochondrial organization. Consistent with this observation, chondrocytes displayed impaired secretion as WGA and collagen II marked intracellular proteins. As with PNA, WGA and collagen II were not uniformly distributed within the *kif5B* mutant cartilage but instead accumulated asymmetrically (Fig 2E and 2F). Interestingly, not all *kif5Ba* single mutants displayed a strong phenotype; in those Dioc6, WGA and collagen II resembled wild-type (S5A Fig). However, *kif5B* compound mutants (*kif5Ba*^*ae12/ae12*^*Kif5Bb*^*ae24/+*^) were indistinguishable from *kif5B* double mutants (S5B Fig); thus, these genotypes were considered to be a single phenotypic group. These observations suggest that Kif5B mediates intracellular organelle distribution and chondrocyte secretion.

**Fig 3:**
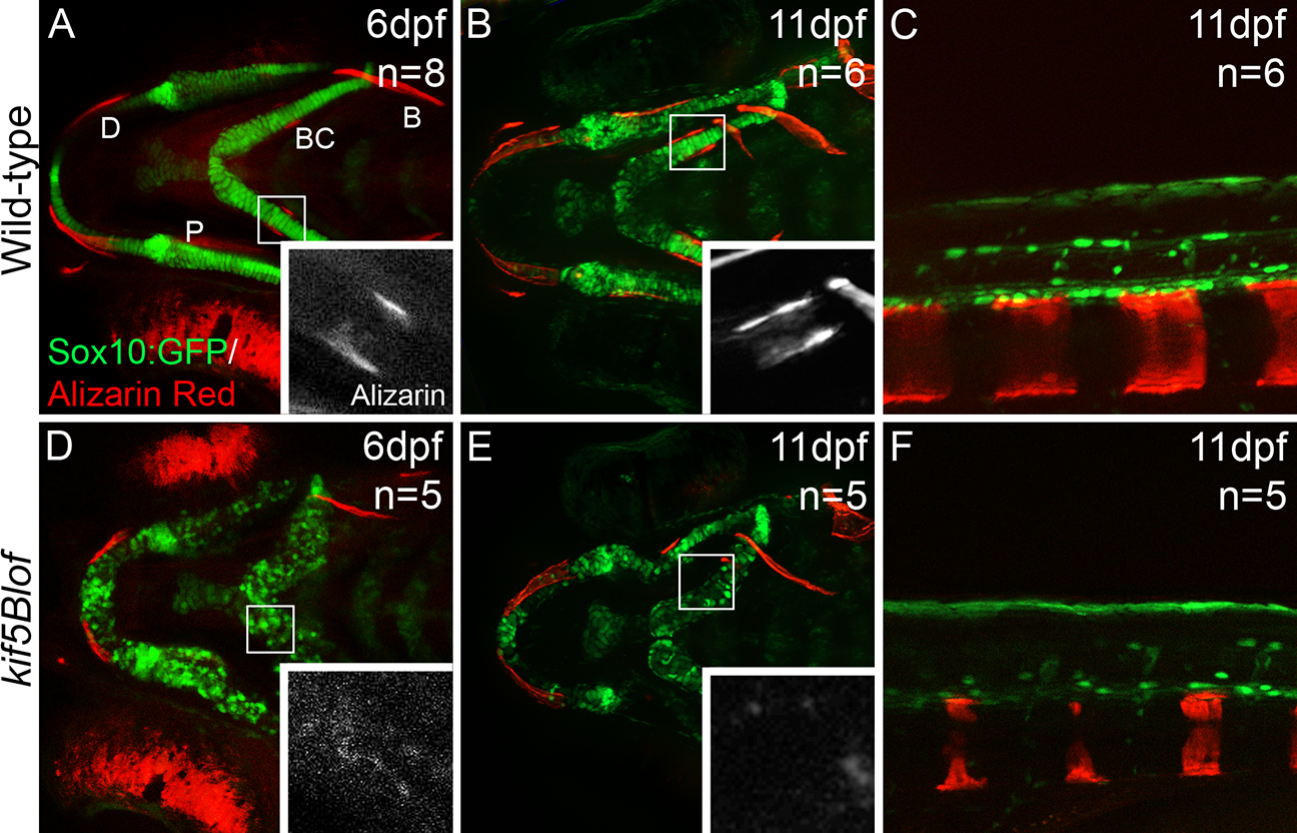
Impeded mineralization in *kif5Blof*. Confocal projections of images of live embryos at 6 dpf (A, D) and 11 dpf (B, C, E, F). GFP stained chondrocytes and Alizarin Red stained mineralized matrix. Intramembranous bones (MD, P and B) show mineralization but were shorter at 6 dpf (A, D). BC, the only endochondral bone at this stage, mineralization was deficient but evidence of mineralization is detectable (insets in D). At 11 dpf membranous and perichondral ossification had progressed in Wt (B) and vertebraes were apparent (C). In *kif5Blof* membranous ossification had also progressed (E) and vertebraes were mineralized (F). However, perichondral ossification was defective as indicated by the absence of BC (E). B: branchiostegal; BC: bone collars; P: entopterygoid; D: dentary.

Because deficient proteoglycan (PG) secretion by chondrocytes can cause premature ossification due to precocious differentiation of perichondrium [7], we examined ossification at 6dpf using Alizarin red. Compared to Wt (Fig 3A), membranous ossification was apparent; however, *kif5Blof* mutants had diminished perichondral ossification (Fig 3D). Bone collars (BC), which develop around Wt cartilage, were not evident in *kif5Blof*, although some mineralization was detectable. Significantly, perichondral ossification was not precocious indicating that either the mutant ECM contains sufficient PG or mutant perichondrium/osteoblasts are nonresponsive or dysfunctional. To distinguish between failed and delayed ossification, we examined bone formation at a later stage, 11 dpf. In Wt, membranous and perichondral ossification had progressed (Fig 3B) and vertebrae were apparent (Fig 3C). In *kif5Blof* membranous ossification had also progressed (Fig 3E), for example the dentary bone was thicker (compare Fig 3D and 3E) and vertebrae (Fig 3F) were apparent although not completely normal. However, the bone collars were not detectable (Fig 3E), suggesting that the mutant cartilage template was unable to support ossification.

### Distinct Kif5B and PCP functions in chondrocyte morphogenesis

**Fig 4:**
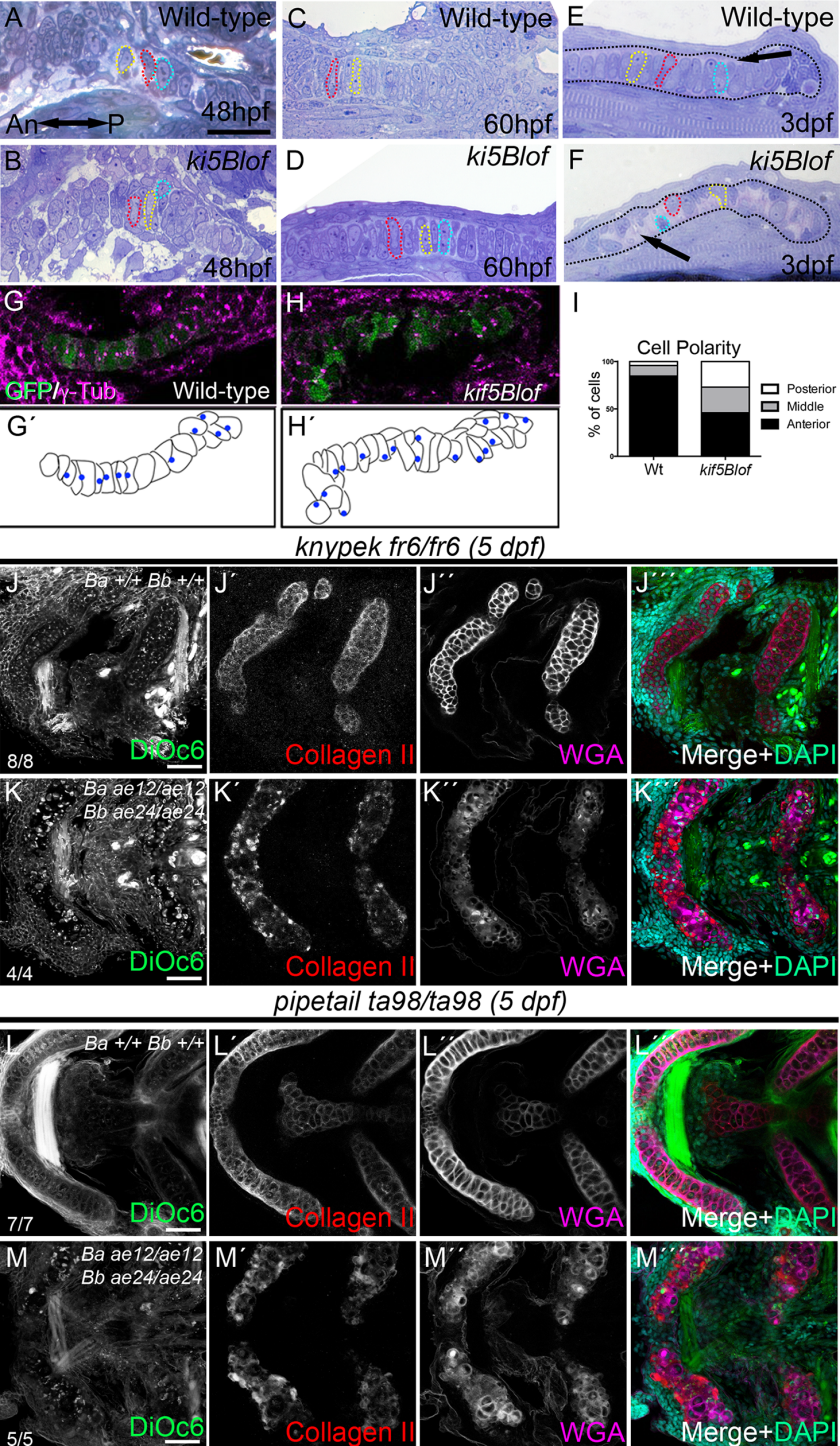
*kif5Blof* defects and interactions with Wnt PCP components. A-F) Semi-thin sections of the trabecular (A, B) and palatoquadrate cartilage (C-F). No differences were detected at 48 hpf (A, B) or 60 hpf (C, D). At 3 dpf, in Wt (E) chondrocytes stack like coins and span the cartilage. However, *kif5Blof* chondrocytes are circular (F), and close to the cartilage border. Some cells are delineated with dashed outlines and in E-F the cartilage limit is drawn. G-I) Analysis of MTOC orientation using γ-Tubulin. In Wt embryos (G, G´) >80% were anteriorly oriented (I). MTOC position was random in *kif5Blof* mutants (H, H´). WT: n = 8; 72cells. *kif5Blof*: n = 5; 63 cells (I). J-K´´´) Confocal images of single *kny* (J-J´´´) or triple mutants (K-K´´´) at 5 dpf. Secretion is normal in single *kny* mutants (J´-J´´´) but stacking is defective (J´´´). Stacking and secretion defects in triple mutants (K-K´´´). L-M´´´) Confocal images of single *ppt* (L-L´´´) or triple mutants (M-M´´´). Secretion is intact in *ppt* single mutants (L´-L´´´) but stacking is disrupted (L´´´). Membrane (M), secretion (M´, M´´) and stacking (M´´´) deficits are evident in triple mutants. ch: ceratohyal; m: Meckel´s; pq: palatoquadrate. Scale bars: A-F: 50μm; G, H: 20μm, J-M´´´: 50μm.

To investigate the subcellular basis of the cartilage defects we conducted histological analysis. At 48hpf there were no overt differences; chondrocytes were unstacked but the trabecular cartilage was morphologically evident in both groups (Fig 4A and 4B). At 60hpf, cell stacking became apparent within cartilage elements, including the palatoquadrate, which were comparable between Wt and mutant groups and elongated chondroctyes spanned the cartilage limits (Fig 4C and 4D). At 3dpf, Wt chondrocytes were elongated and stacked like coins (Fig 4E) with ECM between the chondrocytes and the perichondrium border (Fig 4E). In *kif5Blof* mutants, cells were circular and occupied one side of the cartilage (Fig 4F), while the space devoid of cells contained ECM (Fig 4F). Cell polarization and cell elongation are critical for cartilage stacking [3, 5, 32]. To determine if the rounded *kif5Blof* cell morphology was associated with impaired polarization, we labeled centrosomes with γTubulin and quantified chondrocyte centrosome position along the anterior-posterior axis [3]. In Wt, more than 80% of centrosomes were anteriorly oriented (Fig 4G, 4G´ and 4I); however, in *kif5Blof* mutants the majority of centrosomes were central or posteriorly oriented (Fig 4H, 4H´ and 4I). These results suggest that MTOCs are not polarized at this stage in *kif5Blof* chondrocytes.

Wnt/PCP signaling modulates chondrocyte stacking and elongation in zebrafish [33] and mouse [34]. To determine whether *kif5Bs* interact with PCP components, we generated triple mutants to investigate the effects of combined loss of *kif5Blof* and *knypek*^*fr6*^ (*glypican4*) or *pipetail*^*ta98*^ (*wnt5b*). In both cases triple mutants had more severe craniofacial defects. As previously reported [33, 35, 36] *kny* or *ppt* single mutant cartilage elements were intact but disorganized (Fig 4J´´´ and 4L´´). In triple mutants the cartilage elements were further shortened and more caudally positioned (Fig 4K´´´ and 4M´´´, quantified in S4A–S4D Fig).

To determine the cellular basis of the more severe *PCP*:*kif5Blof* mutant phenotypes DiOC6, collagen II and WGA were examined. DiOC6 in *kny* (Fig 4J–J´´´) or *ppt* (Fig 4L–4L´´´) single mutants resembled Wt, and no secretion defects were detected although mutant cells were round and unstacked (Fig 4J´–4J´´´ and 4L´–4L´´´). Triple mutants were also disorganized, with round unstacked cells like PCP mutants. In addition, collagen II and WGA secretion were impaired like *kif5Blof* (Fig 4K–4K´´´ and 4M–4M´´´). Moreover, although injection of *fgf3* mRNA has been reported to rescue some zebrafish PCP mutants [35] it did not rescue *kif5Blof* (n = 19). Therefore, we conclude the more severe craniofacial defects of *PCP*:*kif5Blof* mutants are due to combined disruption of distinct aspects of chondrocyte morphogenesis that are regulated by PCP and Kif5Bs.

### *Kif5Bs* promote chondrocyte maintenance

**Fig 5:**
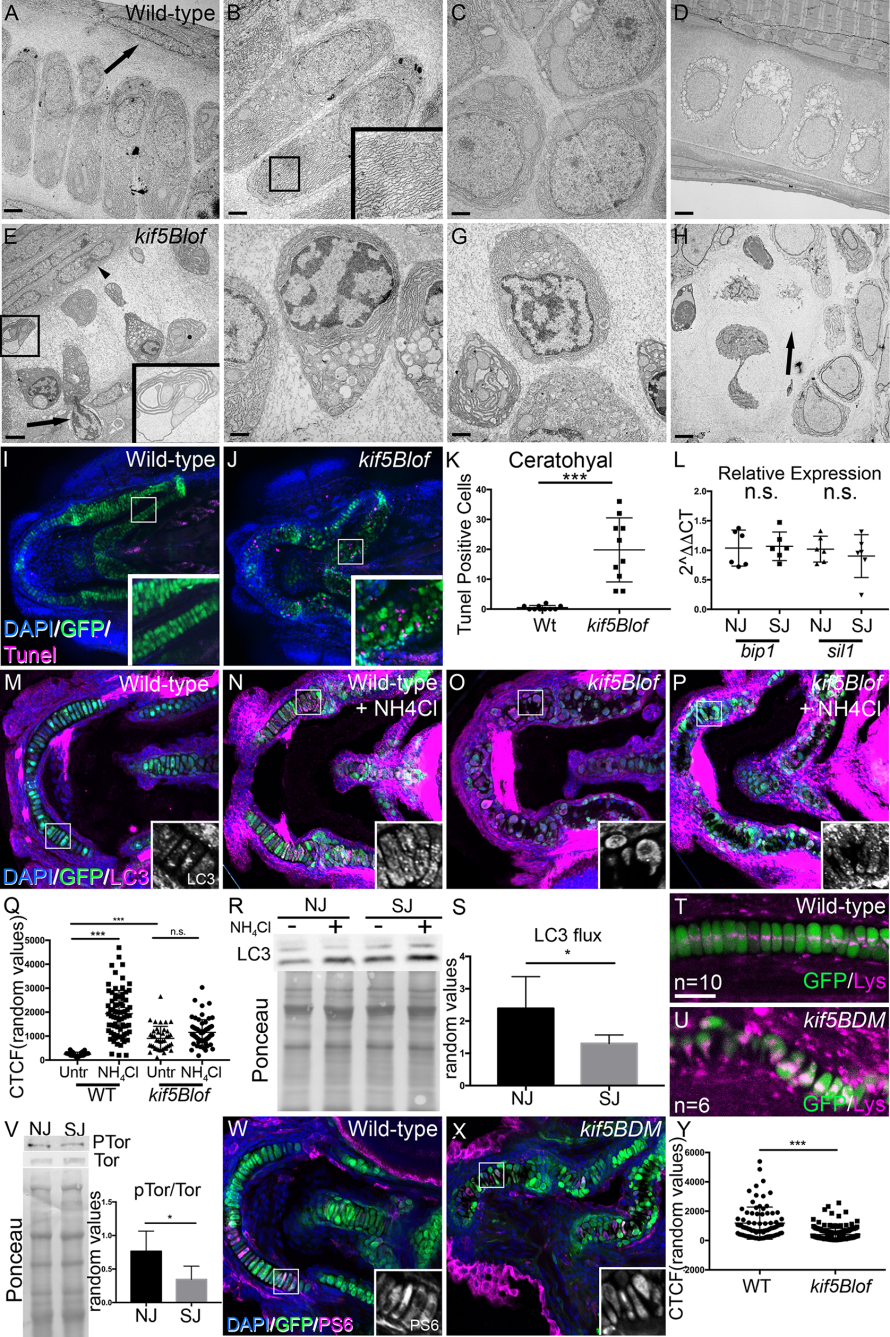
Autophagy and chondrocyte maintenance in *kif5Blof* cartilage. A-D) Electron microscopy of the Wt palatoquadrate. At 3 dpf Wt rER and mitochondria are apparent within stacked chondrocytes (A, B and inset). In the proliferative region (PR), cells are round (C). Cartilage is dense near the perichondrium (arrow in A). At 5 dpf, Wt chondrocytes are hypertrophic with large vacuoles but are stacked (D). E-H) At 3 dpf, *kif5Blof* cartilage contains small, round highly vacuolated cells (E, F and inset) with pronounced rER expansion (E and inset). Both rER and vacuoles appear to contain cellular components (F and inset in E). In the PR, overall chondrocyte morphology is more normal, but with the previously mentioned intracellular characteristics (G). Nuclei are patchy and distributed towards the cartilage border (E, F). Cells are extruded from the cartilage (arrow in E) and the region near the perichondrium is disorganized (arrowhead in E). At 5 dpf, degeneration persists and cartilage “ghosts” remain (arrow in H). I, J, K) Tunel (apoptotic) cells were rare in wild-type embryos, but are abundant in *kif5Blof* mutants. Tunel-positive cells in the ceratohyal (10 embryos were quantified/group). L) ER stress marker (*bip1* and *sil1)* levels. Scale bar: A, D, E, H: 2μm; B, C, F, G: 1μm. n.s. no significant; *** p<0.001 (Student T test). M-S) At 4 dpf in basal conditions, Wt embryos show LC3 positive puncta in chondrocytes (M), which increases after exposure to NH_4_Cl (N). Basal LC3 staining is higher in *kif5Blof* mutants (O) and increases less after drug exposure (P). LC3 fluorescence intensity quantification (5–10 cells in at least 10 embryos/group) (Q, ANOVA). LC3 flux as measured by western blot in embryos with normal jaws and with short jaws (R, LC3 protein and Ponceau staining to reveal total protein loaded, S, quantification, Student T test). I, J) Lysotracker (magenta) stains acidic cellular compartments, including lysosomes. In Wt chondrocytes, several puncta are observed near the nucleus (T). Lysotracker accumulation in *kif5Blof* mutants (U). V) Reduced Tor activation in embryos with short jaws (V, P-Tor and Tor western blot and Ponceau staining to assess total protein loaded, Student T test, n = 4). W-Y) Phospho-S6 Ribosomal Protein (PS6) abundance in chondrocytes of Wt embryos (W) and in *kif5blof* mutants (X), quantified in Y (At least 5–10 cells in at least 10 embryos, Student T test). NJ: normal jaw (Wt), SJ: short jaw (*kif5Ba*^*ae12/ae12*^*Kif5Bb*^*ae24/+*^ and *kif5Ba*^*ae12/ae12*^*Kif5Bb*^*ae24/ae24*^).

In mammals endochondral bone formation requires replacement of calcified hypertrophic chondrocytes with bone [1, 37–39], a process for which several mechanisms have been proposed. In one model, hypertrophic chondrocytes are thought to transform into bone cells [40]. In another, asymmetric division of hypertrophic chondrocytes generates two daughters, one is fated to die and the second becomes an osteoblast [41]. Finally, it has been proposed that terminally differentiated chondrocytes undergo PCD [42, 43]. Thus in mammals removal of the cartilage template during development and transformation of hypertrophic chondrocytes to osteoblast fate during injury repair are essential to preserve tissue architecture. In contrast, in teleost fish the cartilage scaffold persists and becomes surrounded by bone during development and self-renewing periostic cells that surround the cartilage produce “repairing chondrocytes” that mature as osteoblasts during injury repair [44, 45]. Accordingly, cartilage deficits would be expected to compromise bones that form or undergo repair in this manner. Because *kif5Blof* chondrocytes initially elongate but subsequently lose polarity by a mechanism distinct from PCP (Fig 4) we conducted ultrastructural analysis to further characterize the cartilage defect. Within the palatoquadrate, abundant stacked rER and large mitochondria were detected in Wt chondrocytes (Fig 5A and 5B) in stacked regions, whereas proliferative zone cells were more circular (Fig 5C). In both regions, *kif5Blof* chondrocytes were more electron dense with numerous large vesicles containing cellular components reminiscent of autophagic vacuoles (Fig 5E–5G and insets). Most *kif5Blof* chondrocytes had patchy chromatin condensations, vacuole accumulation, and compartmentalization of other organelles within an expanded rER (Fig 5F). An electron dense border of elongated cells abutted the Wt cartilage and adjacent perichondrium (Fig 5A). In contrast, at this stage the perichondrium became disorganized in *kif5Blof* mutants and extrusion of dying chondrocytes was evident (Fig 5F). Later, at 5 dpf, Wt chondrocytes maintained their elongated and stacked arrangement as they became hypertrophic with numerous vacuoles (Fig 5D). In *kif5Blof*, at this stage, matrix remained throughout the mostly hypertrophic chondrocyte depleted cartilage remnant (Fig 5H).

To investigate whether *kif5Blof* cells were indeed dying we performed Tunel staining at 4 dpf (Fig 5I–5K). Whereas Wt cartilage contained very few Tunel-positive chondrocytes, numerous Tunel-positive chondrocytes were detected in *kif5Blof* cartilage (ceratohyal quantified in Fig 5K). Next we investigated whether the expanded rER and death of hypertrophic chondrocytes in *kif5Blof* was associated with elevated ER stress. qRT-PCR analyses of ER stress markers, *bip1* and *sil1*, revealed no significant differences between Wt and mutants (Fig 5L). Therefore, although the ER expands dramatically this does not seem to trigger ER stress based on the markers examined.

Autophagic cell death has been implicated in cartilage remodeling [46]; therefore, we examined autophagy indicators previously used in zebrafish [47, 48] by immunohistochemistry and western blot (Fig 5M–5S). The lipidated form of LC3 (LC3-II) is specific to autophagic structures and indicates autophagic activity. Variably sized LC3 puncta were detected within Wt chondroctyes (Fig 5M and insert). In contrast, LC3 was abundant and within large patches in *kif5Blof* chondrocytes (Fig 5O and 5Q). The degree of LC3-II accumulation following treatment with NH_4_Cl, a lysosomal inhibitor, reflects LC3 flux, and thus autophagic activity [49]. After NH_4_Cl treatment, LC3 increased in Wt (Fig 5N and 5Q), but not *kif5Blof* chondrocytes, which have higher basal LC3 (Fig 5P and 5Q). Quantification of western blots to determine the LC3 flux revealed a significantly higher flux in Wt embryos compared to mutants (Fig 5R–5S). Consistent with the increased basal LC3 staining in *kif5Blof*, examination of lysotracker *in vivo* suggested increased or larger lysosomes (magenta in Fig 5T and 5U). In Wt, discrete LysoTracker puncta were detected adjacent to the nucleus (Fig 5T). In contrast, large LysoTracker-positive aggregates were apparent in *kif5Blof* chondrocytes (Fig 5U). Because some kinesins have been shown to modulate the position of lysosomes, which in turn effects mTOR pathway activity, we examined this negative regulator of autophagy [50]. Consistent with the elevated LC3, western blot analysis revealed reduced mTor pathway activation in *kif5Blof* (Fig 5V), Moreover, phosphorylated S6 ribosomal protein (PS6), a downstream component of Tor activation was weaker in *kif5Blof* chondrocytes (Fig 5W–5Y). Cumulatively, these EM and marker analyses and the impeded mineralization of perichondral bones in mutants suggest that deregulated autophagy underlies the *kif5Blof* cartilage defects.

The *kif5Blof* cartilage phenotypes are reminiscent of those described for MMP14 mutant mice, in that both lead to cartilage devoid of hypertrophic chondrocytes [51]. Given the phenotypic similarities, that metalloproteinases are crucial for cartilage remodeling before mineralization [52–55], and that Kif5B mediates MMP14 delivery to the macrophage cell surface [56], Kif5B could promote chondrocyte maintenance via MMP14. However, no significant differences in total levels or distribution of MMP14 were observed in *kif5Blof* cartilage (S6A–S6C Fig). Moreover, we were unable to phenocopy *kif5Blof* phenotypes with MMP inhibitors (S6D–S6F Fig). Thus, whether *kif5B* acts through MMPs to promote chondrocyte maintenance remains to be determined.

### Cell autonomous and non-cell autonomous effects of *kif5Blof*

**Fig 6:**
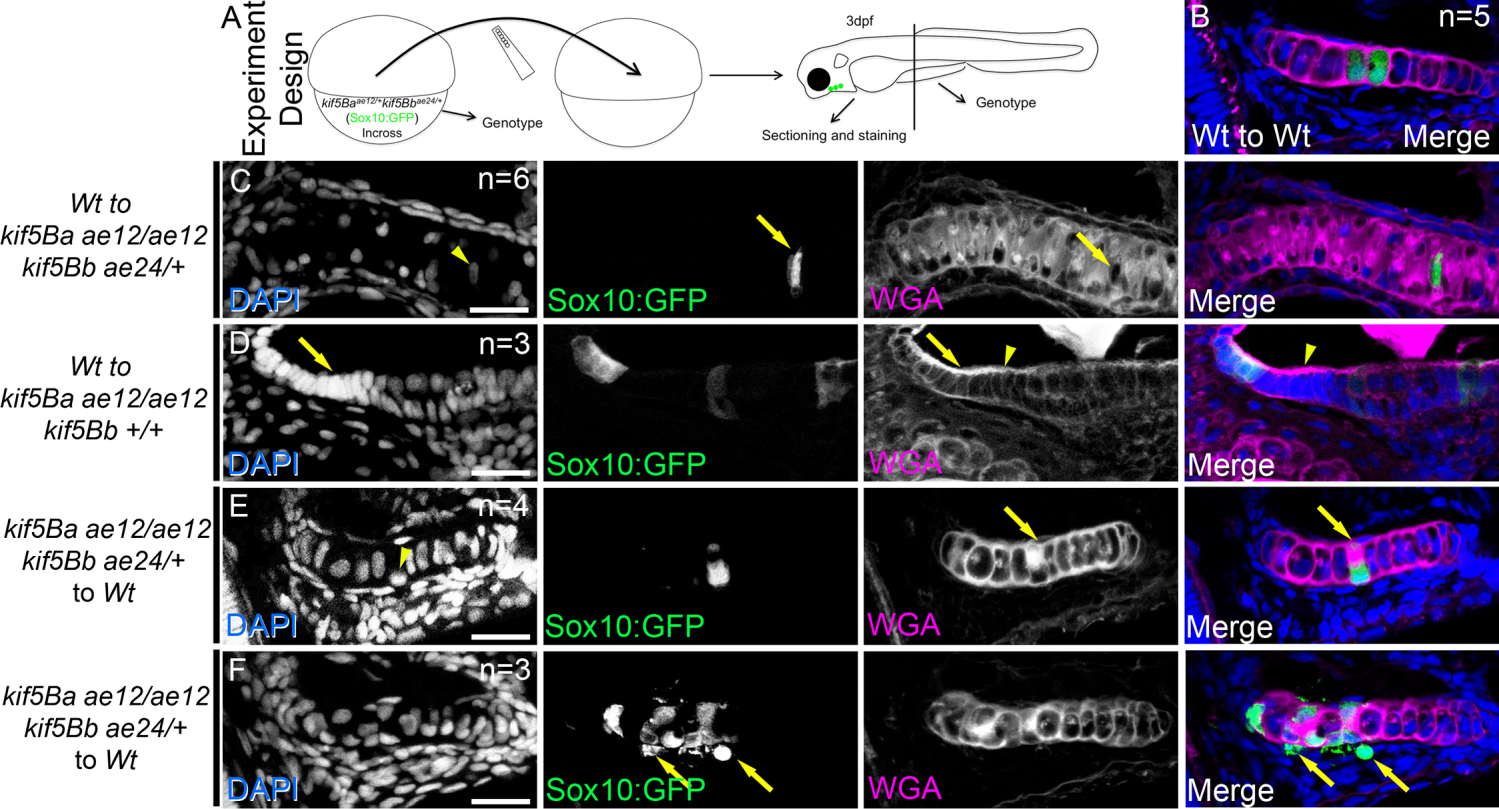
Cell autonomous and cell-nonautonomous *kif5B* effects. A) Cartoon depicts the experimental design. B-F) Confocal images of sectioned palatoquadrate cartilage at 3 dpf. WT cells (GFP+) transplanted to Wt embryos integrate and secrete matrix (B). Single Wt cells (GFP+) in *kif5Blof* elongate, secrete and stack (arrows in C), and the nucleus is centrally positioned compared to mutants (arrowhead in C). Several Wt cells (GFP+) rescue cell stacking in nearby mutant neighbors (arrows in D). Rescue correlates with strong WGA expression along the cartilage border (arrowheads in D). Defects of single *kif5Blof* cells (GFP+) persist in Wt hosts (E) including secretion (arrows in E) and nuclear position (arrowhead in E), although the cells partially elongate. Groups of *kif5Blof* cells (GFP+) in Wt hosts (F) did not stack or elongate and had secretion deficits. Cells are extruded from the cartilage (arrows in F). Successful transplants: Wt to Wt: 13; Wt to *kif5Ba*^*ae12/ae12*^*kif5Bb*^*+/+*^: 2; Wt to *kif5Ba*^*ae12/ae12*^*kif5Bb*^*ae24/+*^: 8; Wt to *kif5Ba*^*ae12/ae12*^*kif5Bb*^*ae24/ae24*^: 2; *kif5Ba*^*ae12/ae12*^*kif5Bb*^*+/+*^ to Wt: 6; *kif5Ba*^*ae12/ae12*^*kif5Bb*^*ae24/+*^ to Wt: 3; *kif5Ba*^*ae12/ae12*^*kif5Bb*^*ae24/ae24*^ to Wt: 0. In the figure, the numbers indicate transplants with similar scenarios for each genotype. Scale bar: 20μm.

We performed transplantations to determine if Kif5B was required cell autonomously in chondrocytes or nonautonomously for ECM integrity or perichondrium interactions (Fig 6A). Cells transplanted from Wt to Wt integrated into host cartilage, were properly elongated, stacked, and produced a uniform ECM (Fig 6B). Similarly, Wt cells in *kif5Blof* mutant hosts elongated and secreted ECM (Fig 6C). Moreover, unlike *kif5Blof* cells whose nuclei were displaced towards the cartilage borders, the nuclei of transplanted Wt were centrally positioned within the elongated cells (Fig 6C). When many Wt cells were transplanted into *kif5B* mutants with intermediate secretion deficits (uniform secretion and robust cartilage borders) the cells elongated and stacked correctly, and strikingly, partially suppressed phenotypes of neighboring mutant cells (Fig 6D), which elongated and stacked. In such cases, suppression correlated with the recovery of a strong WGA boundary between the chondrocytes and the perichondrium. This group effect was not observed when secretion defects were severe (polarized accumulation of proteins and diffuse border).

When mutant cells were transplanted into Wt (Fig 6E and 6F) the cells integrated into the cartilage, and when present as single cells partially elongated although secretion remained polarized (Fig 6E). Moreover, mutant nuclei occupied one side of the cell. When many mutant cells were transplanted into a Wt host their secretion and stacking were abnormal (Fig 6F). Moreover, some mutant cells with defective secretion were outside of the cartilage. We were unable to recover cells transplanted from *kif5Ba*:*Bb* double mutants, although GFP+ cells were detected in other non-cartilage cell types that express Sox10 (n = 11 *kif5Ba*^*ae12/ae12*^*Kif5Bb*^*ae24/ae24*^ donors). The absence of *kif5Ba*^*ae12/ae12*^*Kif5Bb*^*ae24/ae24*^ cells in the cartilage of WT hosts could indicate that double mutant cells were outcompeted by Wt cells, or as suggested by EM and tunel staining, that if *kif5Ba*^*ae12/ae12*^*Kif5Bb*^*ae24/ae24*^ cells were incorporated the cells died and were extruded. Taken together our chimeric analysis indicates that Kif5B is required cell autonomously for secretion and nuclear position. However, Kif5B function has cell non-autonomous consequences, likely ECM-associated as only mutant cells that were surrounded by a normal chrondrocyte:perichondrium border, likely provided by the neighboring WT cells, were elongated.

### Kif5Ba but not Kif5Aa rescues *kif5Blof* chondrocytes

**Fig 7:**
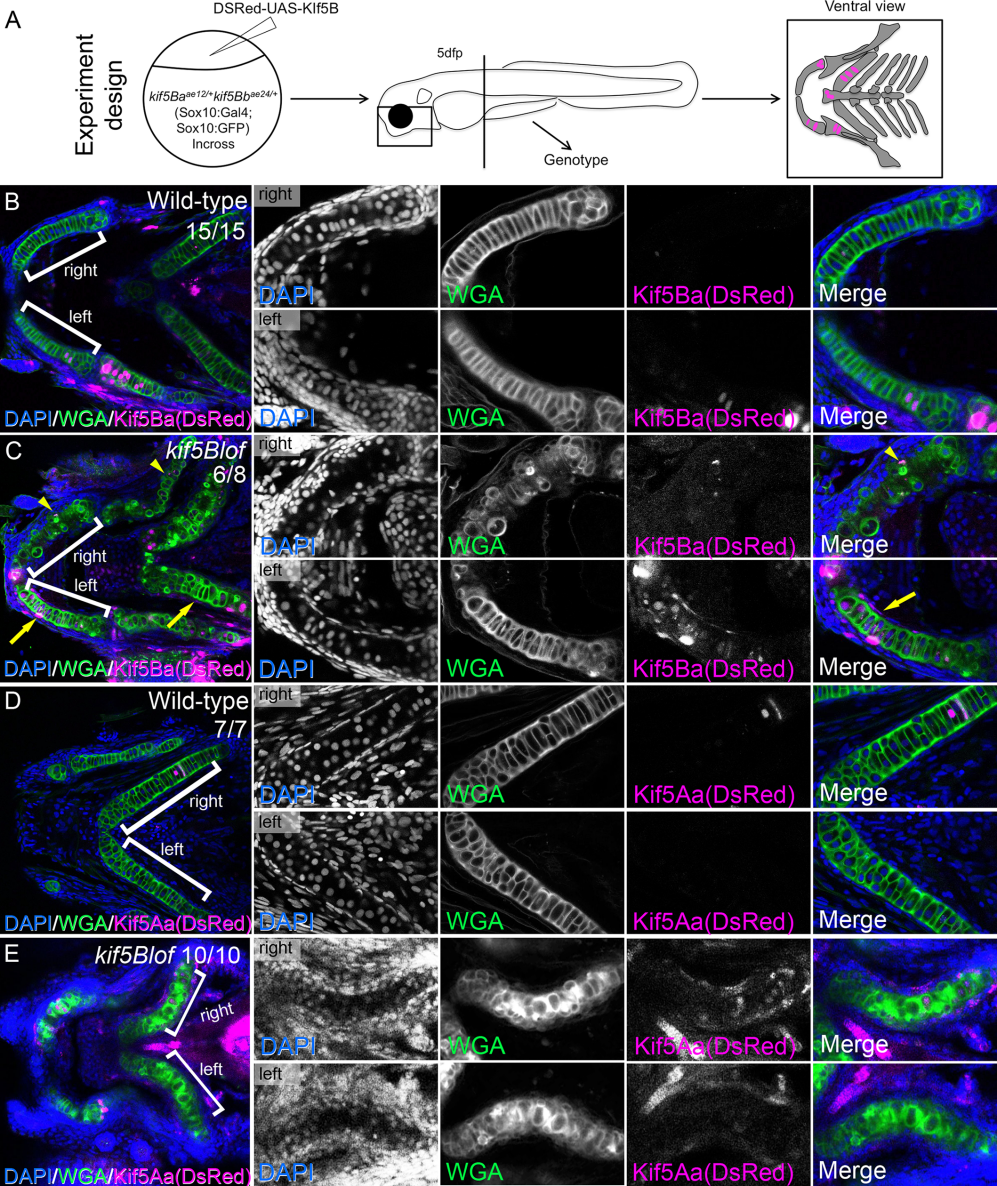
Rescue of *kif5Blof* by Kif5Ba but not Kif5Aa in cartilage. A) Cartoon depicts experimental design. B-E) Kif5Ba or Kif5AaOE did not cause phenotypes in Wt (B, D). Kif5BaOE in *kif5Blof* mutants rescues the phenotype (C, compare right to left). Chondrocytes on the rescued side were stacked and elongated with proper secretion. Rescue improved when both chondrocytes and perichondrium expressed *kif5Ba* (arrows in C) versus chondrocytes alone (arrowheads in C). Overexpression of the neural specific *kif5Aa* did not rescue *kif5Blof* phenotypes (E).

Previously we have used the Gal4/UAS system to rescue *kif5Aa* mutant axons [57]. Here we overexpressed *kif5Ba* specifically in chondrocytes using a *sox10*:*Gal4* line (Fig 7A). We established a compound transgenic line *kif5Ba*^*ae12/+*^*kif5Bb*^*ae24/+*^*;sox10*:*Gal4* and injected the dual expression plasmid *DSRed-UAS-kif5Ba* or *DSRed-UAS-kif5Aa* [57]. This approach yielded embryos mosaically overexpressing *kif5Ba* (Kif5BaOE) (Fig 7B and 7C) or a related Kinesin-1 that is normally restricted to the nervous system, *kif5Aa* [28] (Fig 7D and 7E). Kif5BaOE in Wt chondrocytes caused no phenotypes (Fig 7B), and Kif5BaOE alone rescued *kif5Blof* phenotype (Fig 7C). Chondrocytes expressing DSRed were elongated, secreted properly and were stacked. However, mutant Meckel´s cartilage lacking *kif5Ba*-overexpressing cells was not rescued–the nuclei abutted the cartilage border, and secretion and stacking remained impaired. Kif5AaOE did not cause any phenotypes in Wt (Fig 7D), and did not rescue *kif5Blof* phenotype (Fig 7E). These results indicate that Kif5Bs have specific functions in chondrocytes that the neuronal Kif5Aa cannot fulfill.

## Discussion

**Fig 8:**
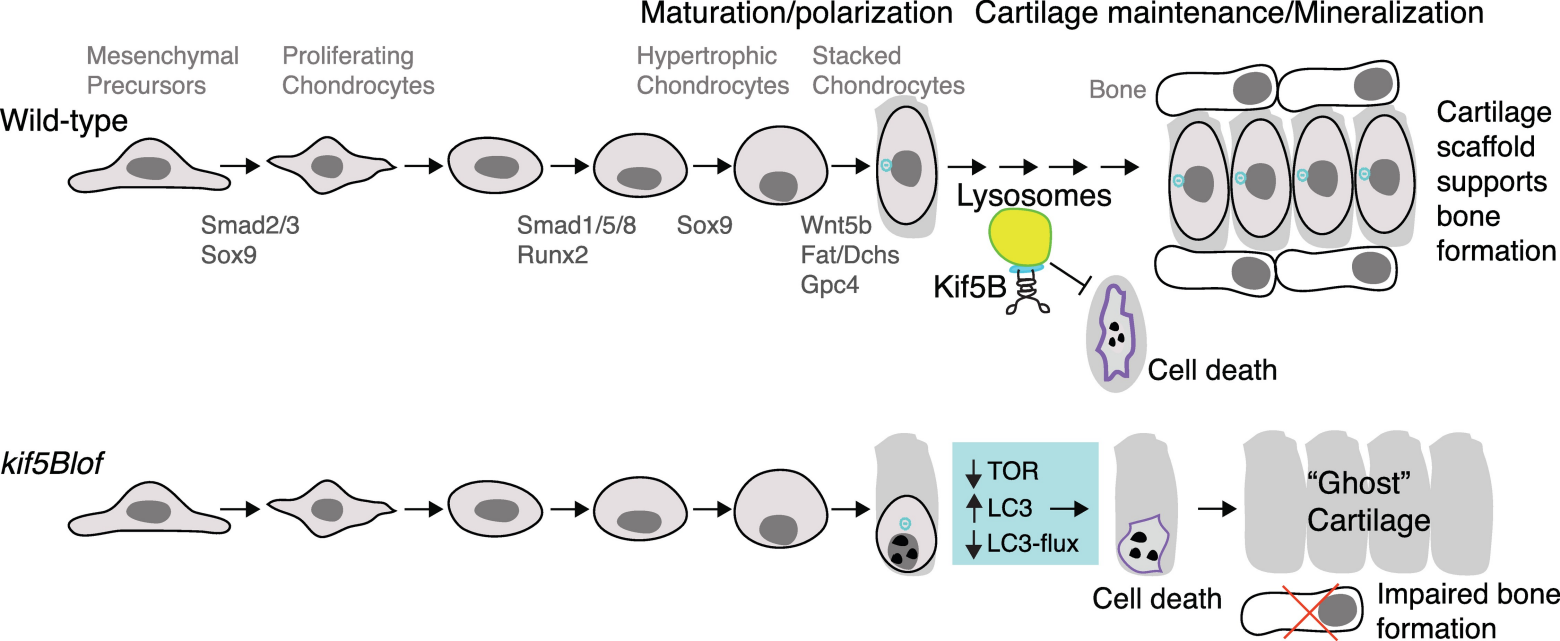
Model for Kif5B roles in cartilage development. Zygotic Kif5Bs are dispensable for the early steps of cartilage specification, PCP-dependent stacking or cell elongation. Zygotic Kif5Bs are required to maintain elongated and polarized chondrocytes and lysosomal function to promote maintenance of hypertrophic chondrocytes and provide a scaffold for bone deposition. Without zygotic Kif5B, TOR activity declines and steady state LC3 increases but lysosomal function (indicated by LC3-flux) declines and mutant chondrocytes are lost, but their ECM persists. This persisting matrix devoid of hypertrophic chondrocytes does not provide sufficient scaffold for optimal perichondral bone formation.

The ossification process is not fully understood [1] but regulation of the cartilage template is essential for vertebrate skeletal morphogenesis. During endochondral bone formation calcified hypertrophic chondrocytes are replaced with bone [1, 37–39]. Several mechanisms have been proposed, including chondrocyte transformation into bone cells [40], asymmetric division of hypertrophic chondrocytes to generate osteoblasts [41], and PCD of terminally differentiated chondrocytes [42, 43]. Distinguishing between these models has been challenging because detection of chondrocyte cell death in normal physiological conditions is rare [13]. However, chondrocyte death is evident in a mouse model of osteoarthritis [46], based on elevated caspase 3 in some cells, suggestive of traditional apoptosis, while other cartilage regions show evidence of autophagic death. The latter PCD has been implicated in removal of terminally differentiated chondrocytes; however, the molecular mechanisms and triggers remain obscure [58–60] and more recent work suggests that terminally differentiated chondrocytes can undergo transformation to an osteoblast fate [40]. In bony fish, the cartilage template persists, forming a scaffold around which bone is deposited [44] (Fig 8). Our data identify cell autonomous functions of Kif5B as an essential regulator of chondrocyte maintenance by a mechanism that involves control of autophagy. Notably, hypertrophic chondrocytes are depleted in *kif5Blof* leaving behind a matrix lacking hypertrophic chondrocytes that consequently impedes skeletal morphogenesis. Kif5B clearly contributes to maintaining differentiated chondrocytes in the cartilage; however, because chondroblast and osteoblast precursors transiently express overlapping markers during development we cannot exclude the possibility that some hypertrophic chondrocytes or perichondrial cells differentiate into repair chondrocytes or osteoblast-like cells with impaired osteogenic potential. Our findings indicate that the chondrocyte microenvironment, ECM and perichondrium, are crucial since large numbers of transplanted Wt cells can rescue intermediate mutant phenotypes, and that persisting hypertrophic chondrocytes are required to provide the scaffold for optimal bone deposition (Fig 8).

Although in this study we have not addressed the mechanism underlying the displacement of the nucleus in chondrocytes, a role for *kif5B* in controlling the position of the nucleus has been described in multinucleate muscle cells in mouse [25] and in *Drosophila* and cultured mammalian cells [61, 62]. It is thus possible that Kif5B also controls the position of the nucleus in chondrocytes. In muscle cells a balance of activity of opposing motors to maintain actin:myosin interactions is thought to maintain nuclear distribution -in mutants or knock down the nuclei collapse to the center of muscle fibers. In cartilage, Kif5B would maintain a centrally positioned nucleus, possibly by opposing other motors as has been shown in other contexts, such that in *kifBlof* the force supplied by Kif5B would be lost and the nucleus would collapse. A second hypothesis would fit the model of Roach et al., which posits that the position of the nucleus changes as the chondrocyte undergoes cell death. Alternatively, the nuclear position in mutants could be secondary to changes in cell morphology or part of an active mechanism to extrude the chondrocytes. Both rescue strategies that we tested (transplantation and supplying Kif5B back to *sox10* expressing cells) were able to restore nuclear position, indicating that this is a cell autonomous function of Kif5B that is likely to be mediated by the Kif5B-dependent mechanisms previously described for maintaining nuclear position in other cell types.

Disrupting secretion is not enough to trigger extensive loss of hypertrophic chondrocytes [6] like *kif5Blof*. Moreover, ER disruption or stress alone, as occurs in zebrafish *bulldog (sec24D)* mutants [8, 63], has not been reported to cause hypertrophic chondrocyte depletion. In culture Kif5B contributes to mitochondrial transport [24] but no obvious cartilage defects were reported, and mitochondria transport defects have not yet been linked to chondrocyte maintenance. Cathepsins are known lysosomal effectors of cell death [64] and function in chondrogenesis [65, 66]. In zebrafish and mammals, interference with lysosomal trafficking disrupts chondrocyte maturation via TGFβ signaling and Cathepsin K, rather than maintenance [65, 67]. Cathepsin D mediates early stages of perichondral ossification [68], and although myopathy was reported in zebrafish morphants no cartilage phenotypes were noted [69]. Cathepsin B is elevated in *in vitro* models of osteoarthritis [70] and promotes early embryonic patterning in zebrafish via BMP signaling, but no jaw phenotypes were noted in mutants [71]. Collectively, the absence of *kif5Blof* like phenotypes in these mutants suggests that Kif5B modulates cell maintenance during chondrogenesis by a mechanism that is distinct from these cathepsins.

Kif5B binds to and functions in lysosomal trafficking of Osteopetrosis Associated Transmembrane Protein 1 (Ostm1), a gene associated with osteopetrosis in humans [72], connecting Kif5B to cartilage/bone development. The roles of lysosomal transport and autophagy during cartilage remodeling remain unclear although autophagy has been linked to osteoarthritis and other bone diseases [73]. Thus, it seems plausible that the high levels of recycling and remodeling that cartilage undergoes require a highly regulated autophagic network. Kif5B has been described to control lysosomal transport [74] and autolysosomal tubulation [75]. In cell culture, Kif2 modulates the activity of mTOR complex (an autophagic inhibitor) by controlling lysosomal subcellular position; only lysosomes distal to the MTOC have high mTOR activity and low autophagy levels while lysosomes proximal to the MTOC and the nucleus cannot activate MTOR and have elevated autophagy [50]. This relationship appears deregulated in *kif5Blof* mutants (Fig 8) with the absence of *kif5B* inducing higher levels of LC3. However, *kif5Blof* mutants are refractory to the lysosomal inhibitor NH_4_Cl, reflecting strong autophagy activation, but blocked autophagosome-lysosome fusion, hallmarks of autophagic cell death [76]. This process is concomitant with low Tor pathway activation and accumulation of lysosomes near the nucleus. Why the cartilage is most affected remains an open question. It is unclear if *kif5B* acts specifically in chondrocytes or more generally controls autophagy in earlier development, in which case the high maternal load likely supplies earlier Kif5B function. Strikingly, overexpression of the related Kinesin-1, Kif5Aa, which is overall highly similar [28] but has a divergent and longer C-terminal tail compared to Kif5Bs [57] does not rescue *kif5Blof* suggesting a specific Kif5B role. Other Kifs may compensate in other tissues or cartilage may uniquely require high levels of cellular recycling at this stage.

Wnt PCP signaling modulates cell polarization, elongation, and stacking during chondrogenesis [33, 77]. Wnt5b ligand and the PCP coreceptor and proteoglycan, Gpc4, regulate cell elongation and stacking, but not secretion during chondrogenesis [4, 5, 33, 35]. We found that zygotic Kif5B was dispensable for these early events, but acts later to maintain cell elongation. Moreover, although supplying *fgf3* mRNA has been reported to rescue some zebrafish PCP mutants [35] it does not rescue *kif5Blof*. Consistent with independent contributions of Kif5Bs and these PCP effectors to chondrogenesis, combined loss of PCP components and *kif5Bs* produced additive phenotypes. Together these molecular and genetic data are consistent with independent roles of Kif5Bs and Wnt PCPs in chondrogenesis. However, due to abundant maternal contribution of *kif5Bs* [26, 28] we cannot exclude a role for maternal Kif5B in earlier PCP-processes.

During normal development mesenchymal progenitors differentiate into chondrocytes that elongate, stack, and later terminally differentiate into hypertrophic cells. These cells eventually support bone deposition through a process that is not fully understood (Fig 8). Without zygotic Kif5B the early specification steps are intact and mutant chondroctyes elongate and stack. However, later they fail to maintain this morphology, collapse, and undergo cell death. Although dying mutant cells are eliminated the cartilage matrix remains as an empty “ghost” cartilage thereby impeding bone formation (Fig 8). Cumulatively, our study reveals an essential role for Kif5B in cell polarization, secretion, autophagy and chondrocyte maintenance. Our findings suggest that Kif5Bs are a novel component acting to preserve differentiated chondrocytes prior to bone mineralization. *kif5Blof* mutants provide a genetically and pharmacologically tractable *in vivo* model to study cartilage development and bone deposition and the mechanisms that promote hypertrophic chondrocyte maintenance.

## Methods

### Animals

Embryos were obtained from natural pairwise matings and reared in standard conditions [78]. Embryos were raised in 1×Embryo Medium (EM) at 28.5°C and staged according to [79]. All procedures and experimental protocols were in accordance with NIH guidelines and approved by the Einstein IACUC (protocol #20140502). To avoid pigmentation, embryos were treated with PTU from 24 hpf onwards.

*Sox10*:*GFP*, *Sox10*:*Gal4* and *pipetail*^*ta98*^ and *Knypek*^*fr6*^ (see Table 1 for genotyping primers) lines were crossed with the *kif5Ba*^*ae12/+*^*kif5Bb*^*ae24/+*^ line to make compound mutants.

**Table 1:** Primers used in this work.

**Genotyping**	
*kif5Ba*^*ae12*^*-F*	GGAGTGCACCATTAAAGTCATGTG
*kif5Ba*^*ae12-*^*R*	GTCGGTGTCAAATATTGAGGTC
*kif5Bb*^*ae21*^*-F*	CATTTCAAGGAGAGGACAGTGTGCTGA
*kif5Bb*^*ae21*^*-R*	AGGTGGTTAGCGGATGCTAATA
*kif5Bb*^*ae22*,*ae23*,*ae24*^*-F*	ATCATCCTCATCATCCTCCTTCTC
*kif5Bb*^*ae22*,*ae23*,*ae24*^*-R*	AGGTGGTTAGCGGATGCTAATA
*pipetail*^*ta98*^*-F*	GATTACTGCCTGCGCAATGAAAC
*pipetail*^*ta98*^*-R*	GGTCTTGAACTGGTCGTACACGCG
*Knypek*^*fr6*^*-F*	GACCAATCAAGGCTTATCTTC
*Knypek*^*fr6*^*-R*	AACTAACAATTAAGGAGGGCTA
*Gal4-F*	CTCCCAAAACCAAAAGGTCTCC
*Gal4-R*	TGAAGCCAATCTATCTGTGACGG
**Mutagenesis**	
*kif5Bb gRNA-F*	TAGGAGAGGACAGTGTGGTCAT
*kif5Bb gRNA-R*	AAACATGCACCACACTGTCCTCT
**qRT-PCR**	
*ef1α-F*	AGCCTGGTATGGTTGTGACCTTCG
*ef1α-R*	CCAAGTTGTTTTCCTTTCCTGCG
*kif5Ba-F*	GACTTTGCACAACCTCAGGAAA
*kif5Ba-R*	GCAGACGCTTCTCCAGTTTAGG
*kif5Bb-F*	TCTCAATGAGGAGCTGGTCAAA
*kif5Bb-R*	GTTTTTCATGCTCCACCTTCAA
*bip-F*	CAGGAAAGAGTAAAACAGCAACCG
*bip-R*	CCGAAATTTTGCTCTCACTGCATC
*sil-F*	CAGGAAAGAGTAAAACAGCAACCG
*sil-R*	CCGAAATTTTGCTCTCACTGCATC
*sox9a-F*	GACCCCTACCTGAAGATGAC
*sox9a-R*	CGCGGAGTCCTCGGACATG
*col2a1-F*	GCTGGATTCACGGACTCTCC
*col2a1-R*	CCTTTGCACCAAGTGACCGG

### Drug treatment

NH_4_Cl (Sigma 254134) was performed as previously described [80]. Embryos were exposed to 100nM of NH_4_Cl in EM for 24 hours from 3 dpf to 4 dpf. After rinsing in EM, embryos were prepared for western blot or staining.

To determine if MMP inhibitors would cause *kif5Blof-like* phentoypes, we exposed Wt (*sox10*:*GFP*) embryos to either the broad-spectrum metalloproteinase inhibitor EDTA (0.5mM and 1mM) or the MMP inhibitor GM6001 (200μ; Santa Cruz, sc-203979) from 48 hpf to 4 dpf with daily changes of the medium and drug daily. Sibling embryos were exposed to DMSO 1% as a control.

### Mutagenesis

*kif5Ba* and *kif5Bb* zebrafish mutants were made using CRISPR-Cas9-mediated mutatgenesis (http://www.addgene.org/crispr/jounglab/CRISPRzebrafish/) [81, 82]. *kif5Bb* mutagenesis was as for *kif5Ba* [26]. *gRNA* primers (Table 1) were annealed and ligated to make *pDR272-kiftBb-gRNA*. Screening was performed using T7 endonuclease assays [81] with genomic DNA [78, 83]. Primers are in Table 1. The *kif5Bb*^*ae21*^ allele yields a smaller fragment upon DdeI digest of PCR products. Similarly, *kif5Bb*^*ae22*^, *kif5Bb*^*ae23*^ and *kif5Bb*^*ae24*^ alleles yield smaller products when PCR products are digested with AhdI, DpnII, and HinfI, respectively.

### UAS plasmids and microinjection

Directional UAS:DsRed;UAS:Kif5 with either Kif5Aa [57] or Kif5Ba [26] were injected at approximately 1 nl of a 10–20 ng/μl solution into single-cell embryos from *kif5Ba*^*ae12/+*^*kif5Bb*^*ae24/+*^*Sox10*:*Gal4* incrosses.

The *fgf3* plasmid was a kind gift from M. Tada (UCL). Plasmid was linearized using BamHI and mRNA was made using T7 RNA polymerase.

### qRT-PCR

Total RNA was extracted from pooled 48 hpf (n = 10) or 5dpf larvae (n = 20–30) using Trizol (Life Technologies, 15596). cDNA was prepared with the SuperScript III Reverse Transcription Kit (Life Technologies, 18080–051). qRT-PCR, reactions were performed in triplicate with SYBR Green Master Mix (Thermo Scientific) using an Eppendorf realplex2 Mastercycler. See Table 1 for primers. The ΔΔCt method was used to quantify expression relative to WT, using *ef1α* [83].

### Histology

For semithin and ultrathin sections, samples were fixed with 2.5% glutaraldehyde, 2% paraformaldehyde in 0.1 M sodium cacodylate buffer, postfixed with 1% osmium tetroxide followed by 2% uranyl acetate, dehydrated through a graded series of ethanol and embedded in LX112 resin (LADD Research Industries, Burlington VT). Ultrathin sections were cut on a Reichert Ultracut UCT, stained with uranyl acetate followed by lead citrate and viewed on a JEOL 1200EX transmission electron microscope at 80kv.

Embryos for cryosectioning were cryoprotected in 15% sucrose in PBS overnight at 4°C and then in 30% sucrose in PBS 2 hours, room temperature. Samples were oriented in OCT using Tissue-Tek cryomolds (25608–924; VWR) and then frozen with liquid nitrogen. Samples were kept at -80°C. 10 μM sections were obtained using a Leica CM 1900 cryostat.

### Stainings

Chromogenic *in situ* hybridization was performed according to established protocols [84]. *sox9a* and *col2a1* probes were generous gifts from T. Schilling. For anti-sense probes, *sox9a* plasmid cDNA was linearized using SmaI, and *col2a1* plasmid cDNA was linearized using Pst1; both ribo-probes were synthesized using Sp6 polymerase and digoxygenin labeled nucleotides.

For whole-mount immunohistochemistry (IHC) [85], embryos were fixed in PFA 4% overnight at 4°C. After washing with PBS 0.1% Triton-x100, they were permeabilized with proteinase K according to the stage. Primary antibody was incubated overnight at 4°C in PBS 0.5%Tx and 5% goat serum, followed by washes with PBS 0.1%Tx and incubation with secondary antibody overnight at 4°C. Nuclei were counterstained with DAPI (1:10,000; D9542, Sigma). Actin was detected using AlexaFluor-658 Phalloidin (1/100 [3]; A12380, Life Technologies), cell secretion was detected using Alexafluor-647 PNA (1/200; L-32460, Molecular Probes) and Alexafluor-640 WGA (1/200; 97065–738, VWR), intracellular membranes were labeled using Di0C6 (5 μg/ml; D273, Invitrogen). Primary antibodies: mouse anti-GFP (1/1000; A11120, Life Technologies), chicken anti-GFP (1/500; A10262, Life Technologies), mouse anti Collagen II (1/100; II-II63, DSHB), mouse anti-γTubulin (1/500; T5326, Sigma), acetylated Tubulin (1/500 T7451), rabbit anti-LC3 (1/100; ab51520, Abcam), rabbit anti-Phospho-S6 Ribosomal Protein (PS6) (1/100, #4858, Cell Signaling). Secondary antibodies: AlexaFluor-488 anti-mouse (1/300; A-11001, Life Technologies), AlexaFluor-488 anti-chicken (1/300; 103-545-155, Jackson), AlexaFluor-568 anti-mouse (1/300; A-11004, Life Technologies), AlexaFluor-568 anti-rabbit (1/300; A-11011, Life Technologies).

For IHC on cryosections [85], samples were rehydrated in PBS and pre-incubated in PBS 0.2% Triton-x100. Primary antibody incubation was overnight at 4°C, followed by PBS washes. Sections were next incubated in secondary antibody 1 hour at room temperature (RT). Sections were mounted in DAPI Fluoromount-G (0100–20, SouthernBiotech) and coverslipped.

Tunel was performed as previously described [85] in whole-mount embryos. Tunel positive cells were quantified using ImageJ.

Staining against MMP14 (Abcam, ab53712) was performed using a TSA amplification system (NEL704A001KT, PerkinElmer) [86].

Alcian Blue staining was performed using an acid-free protocol [87]. Embryos were fixed in 4% PFA in PBS for 2 hours at RT. After several washes in PBS, samples were dehydrated in Ethanol. Cartilage was stained using 0.2% Alcian Blue (862; Anatech) dissolved in 70% ethanol/50 mM MgCl_2_ for 2 hours at RT. Embryos were rehydrated and clarified using 1% KOH solution before imaging.

Alizarin red was conducted *in vivo* for *Sox10*:*GFP* larvae [88] at 6 dpf and 11 dpf. Larvae were incubated in 30 ml EM with 200 μl 0.5% Alizarin Red solution (A475-03, J.T.Baker) for 3 hours before imaging.

### Cellular transplantations

Cellular transplants were performed as previously reported [3]. Donor (*sox10*:*GFP*) embryos were injected with 3% Rhodamine-Dextran and then dechorinated manually. At 30% epiboly, cells were transferred from labeled donors to hosts with a capillary needle. Embryos were raised in 1xRinger solution with Penicillin/Strep until 24 hpf. Then embryos were transferred into 1xEM. Host embryos were fixed at 4 dpf and prepared for sectioning. The donors (shield-60% epiboly) were lysed and genotyped using the primers and the digestion described in the mutagenesis section above.

### Western blot

Western blot was performed as previously described [89]. 25 embryos (1 group) were manually deyolked at 5 dpf and then resuspended in 50 μl SDS 2x buffer. Embryos were classified into groups based on the phenotype of the Sox10:GFP cells (Normal jaw: Wt, Short jaw: *kif5Ba*^*ae12/ae12*^*Kif5Bb*^*ae24/+*^ and *kif5Ba*^*ae12/ae12*^*Kif5Bb*^*ae24/ae24*^ mutants). 10μl per sample was loaded in a 12% SDS-page gel, proteins were then transfered to PVZ membranes. Rabbit anti-Phospho-mTOR (Ser2448) (1/1000, #2971, Cell Signalling), rabbit anti-mTOR (1/1000, #2972, Cell Signalling), rabbit anti-LC3 (1/1000, ab51520, Abcam) and mouse MMP14 antibody (1/1000) was followed by HRP anti-rabbit (1/5000; Milipore, 12–348). Ponceau staining was used as a loading control and to normalize MMP14 expression. Membranes were imaged in G:BOX Chemi XX6 (Syngene). Signals were quantified with Image J.

### Image acquisition and processing

Live embryos or *in situ* hybridization stainings were imaged using an Olympus SZ61 dissecting microscope with a high-resolution digital camera (model S97809, Olympus America) and Picture Frame 3.0 software (Optronics).

Semi-thin sections and flat-mounted embryos from Alcian blue staining were imaged using a Zeiss Axioskop2 plus microscope with a Zeiss AxioCam MRc camera and Zeiss AxioVision Rel. 4.6 software.

Live embryos, whole-mount and sections immunohistochemistry were visualized using an inverted Leica Sp2 AOBS confocal microscope using either 40x or 63x oil-immersion objective. For wholemount, embryos were embedded in 1% low-melt agarose (BP165-25, Fisher) prior to imaging. Images were acquired using Leica software.

### Image analysis and statistics

Jaw protrusion was determined from dorsal views of 6dpf larvae by measuring the length of tissue extended beyond the eyes using ImageJ. Measurements of the cartilage elements were determined from ventral views of alcian blue stained larvae using ImageJ.

For the autophagic markers LC3 and PS6 the intensity of the fluorescence was quantified as Corrected Total Cell Fluorescence (CTCF = Integrated Density—(Area of selected cell X Mean fluorescence of background readings)) using ImageJ. CTCF was quantified in individual chondrocytes using Sox10:GFP to determine cellular outlines.

Z-stacks were analyzed in ImageJ and figures were prepared using Adobe Photoshop CS3 where brightness and contrast were adjusted to reflect that observed with the microscope.

Statistics were performed using Prism7 for Mac OSX, version 6.0c.

## Supporting information

S1 Fig Muscle phenotypes of *kif5Blof* mutants. A-D) Ventral view of the anterior region of zebrafish at 5 dpf. Muscle fibers are shorter than Wt, and broken muscle fibers are detected along the body axis (arrows in C and D). This phenotype was not observed in *kif5Ba* single mutants (B). E-H) Lateral views of the tail at 5 dpf; broken muscle fibers are observed in *kif5Blof* mutants (arrows in G and H). I-L) Electron microscopy of the ocular muscle at 3 dpf (I, J) and 5 dpf (K, L). No disruption of muscle ultrastructure was apparent at 3 dpf (I, J). However, at 5 dpf the M-line of the sarcomere is diminished in *kif5Blof* mutants (I, J and insets).
(TIF)

S2 Fig Central nervous system is not altered in *kif5Blof* mutants. Ventral (A, D) or lateral (B, E) view of the head and lateral view of the tail (C, F) of Acetylated tubulin staining in Wt (A-C) and *kif5Blof* mutants (D-F).
(TIF)

S3 Fig *sox9a* and *col2a1* expression levels in *kif5B* mutants resembles Wt. qRT-PCR for *col2a1* (A) and *sox9a*(B) showed no differences between Wt and *Kif5B* mutants. n.s. No significant after Student T test. Three different batches of embryos were included in each group.
(TIF)

S4 Fig Measurements of the cartilage elements in *kif5B*, *knypek* and *pipetail* mutants. Quantification of the Ch angle (A), Ch length (B), the distance between Meckel´s and ceratohyal (C), and the extension of the ceratohyal cartilage along the antero-posterior axis. In *kif5Blof* mutants (second column), the angle was wider, the length was shorter, the M-Ch distance was shorter and the AP extension was reduced. In *knypek* mutants (third column) the angle was wider, the length and the AP extension were shorter, and these conditions were aggrevated in the triple mutants. In *pipetail* mutants (fourth column) the angle was wider, the length and the AP extension were shorter, and the ceratohyal was shorter in triple mutants. One-way ANOVA * p<0.05, ** p<0.01, *** p<0.001. AP: antero-posterior axis; Ch: ceratohyal; M: Meckel´s.
(TIF)

S5 Fig Secretion defects in *kif5B* compound mutants. Some *kif5B* single mutants **(***kif5Ba*^*ae12/ae12*^*Kif5Bb*^*+/+*^) resembled Wt in terms of membrane distribution and secretion (A). *kif5B* compound mutant (*kif5Ba*^*ae12/ae12*^*Kif5Bb*^*ae24/+*^) phenotypes were fully penetrant and indistinguishable from double mutants.
(TIF)

S6 Fig MMP14 distribution is not altered in *kif5Blof* mutants. Chondrocytes and perichondrium are positive for MMP14 in Wt (A) and *kif5Blof* (B). Western blot reveals no significant changes [90] in MMP14 levels (C). Scale bar: 1 μm. D-G) Wt embryos show no *kif5Blof-like* phenotypes when exposed to broad-spectrum metalloproteinase inhibitor (E, F) or the MMP inhibitor GM6001 (G), although some cells were extruded from the cartilage at high concentrations of EDTA (arrow in F).
(TIF)
