# Correction: Kinesin-1 promotes chondrocyte maintenance during skeletal morphogenesis

**DOI:** 10.1371/journal.pgen.1007099

**Published:** 2017-11-15

**Authors:** Adrian Santos-Ledo, Marina Garcia-Macia, Philip D. Campbell, Marta Gronska, Florence L. Marlow

In Table 1, the incorrect primer sequences are shown for *bip-F* and *bip-R*. The sequence for *bip-F* should read *bip-F*: AGTGATTGGGATCGACCTTG and the sequence for *bip-R* should read *bip-R*: AGCTGGATGTGAGGCTTGTT. Please see the corrected Table 1 here.

**Table 1:** Primers used in this work.

**Genotyping**	
*kif5Ba*^*ae12*^*-F*	GGAGTGCACCATTAAAGTCATGTG
*kif5Ba*^*ae12-*^*R*	GTCGGTGTCAAATATTGAGGTC
*kif5Bb*^*ae21*^*-F*	CATTTCAAGGAGAGGACAGTGTGCTGA
*kif5Bb*^*ae21*^*-R*	AGGTGGTTAGCGGATGCTAATA
*kif5Bb*^*ae22*,*ae23*,*ae24*^*-F*	ATCATCCTCATCATCCTCCTTCTC
*kif5Bb*^*ae22*,*ae23*,*ae24*^*-R*	AGGTGGTTAGCGGATGCTAATA
*pipetail*^*ta98*^*-F*	GATTACTGCCTGCGCAATGAAAC
*pipetail*^*ta98*^*-R*	GGTCTTGAACTGGTCGTACACGCG
*Knypek*^*fr6*^*-F*	GACCAATCAAGGCTTATCTTC
*Knypek*^*fr6*^*-R*	AACTAACAATTAAGGAGGGCTA
*Gal4-F*	CTCCCAAAACCAAAAGGTCTCC
*Gal4-R*	TGAAGCCAATCTATCTGTGACGG
**Mutagenesis**	
*kif5Bb gRNA-F*	TAGGAGAGGACAGTGTGGTCAT
*kif5Bb gRNA-R*	AAACATGCACCACACTGTCCTCT
**qRT-PCR**	
*ef1α-F*	AGCCTGGTATGGTTGTGACCTTCG
*ef1α-R*	CCAAGTTGTTTTCCTTTCCTGCG
*kif5Ba-F*	GACTTTGCACAACCTCAGGAAA
*kif5Ba-R*	GCAGACGCTTCTCCAGTTTAGG
*kif5Bb-F*	TCTCAATGAGGAGCTGGTCAAA
*kif5Bb-R*	GTTTTTCATGCTCCACCTTCAA
*bip-F*	AGTGATTGGGATCGACCTTG
*bip-R*	AGCTGGATGTGAGGCTTGTT
*sil-F*	CAGGAAAGAGTAAAACAGCAACCG
*sil-R*	CCGAAATTTTGCTCTCACTGCATC
*sox9a-F*	GACCCCTACCTGAAGATGAC
*sox9a-R*	CGCGGAGTCCTCGGACATG
*col2a1-F*	GCTGGATTCACGGACTCTCC
*col2a1-R*	CCTTTGCACCAAGTGACCGG
