# Supplementary figures and images for: Kinesin-1 promotes chondrocyte maintenance during skeletal morphogenesis
Source: PLoS Genet. 2017 Jul 17;13(7):e1006918. doi: 10.1371/journal.pgen.1006918 (PMC5536392; doi:10.1371/journal.pgen.1006918)

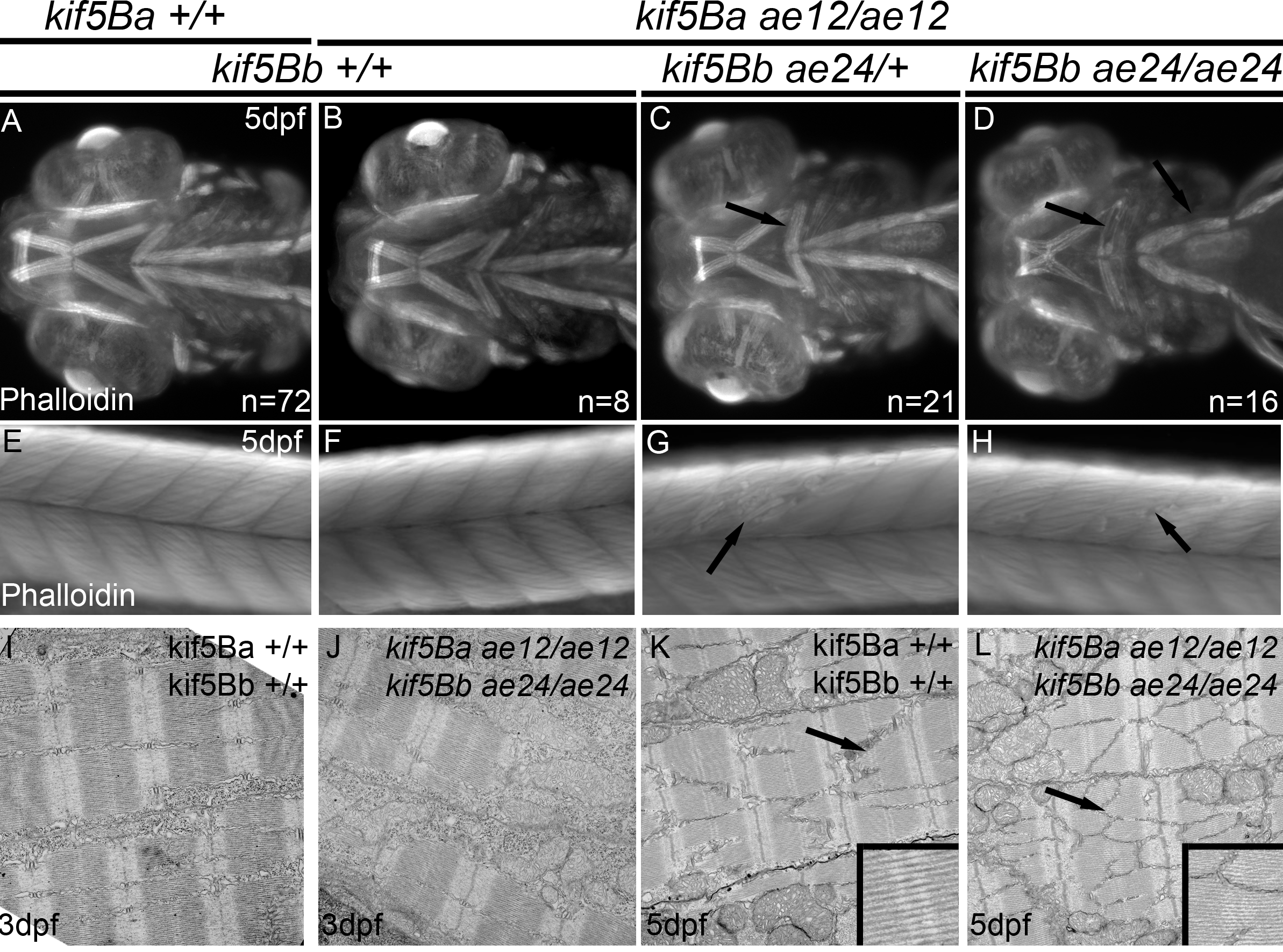

Supplement: S1 Fig — A-D) Ventral view of the anterior region of zebrafish at 5 dpf. Muscle fibers are shorter than Wt, and broken muscle fibers are detected along the body axis (arrows in C and D). This phenotype was not observed in kif5Ba single mutants (B). E-H) Lateral views of the tail at 5 dpf; broken muscle fibers are observed in kif5Blof mutants (arrows in G and H). I-L) Electron microscopy of the ocular muscle at 3 dpf (I, J) and 5 dpf (K, L). No disruption of muscle ultrastructure was apparent at 3 dpf (I, J). However, at 5 dpf the M-line of the sarcomere is diminished in kif5Blof mutants (I, J and insets). (TIF) [file pgen.1006918.s001.tif]

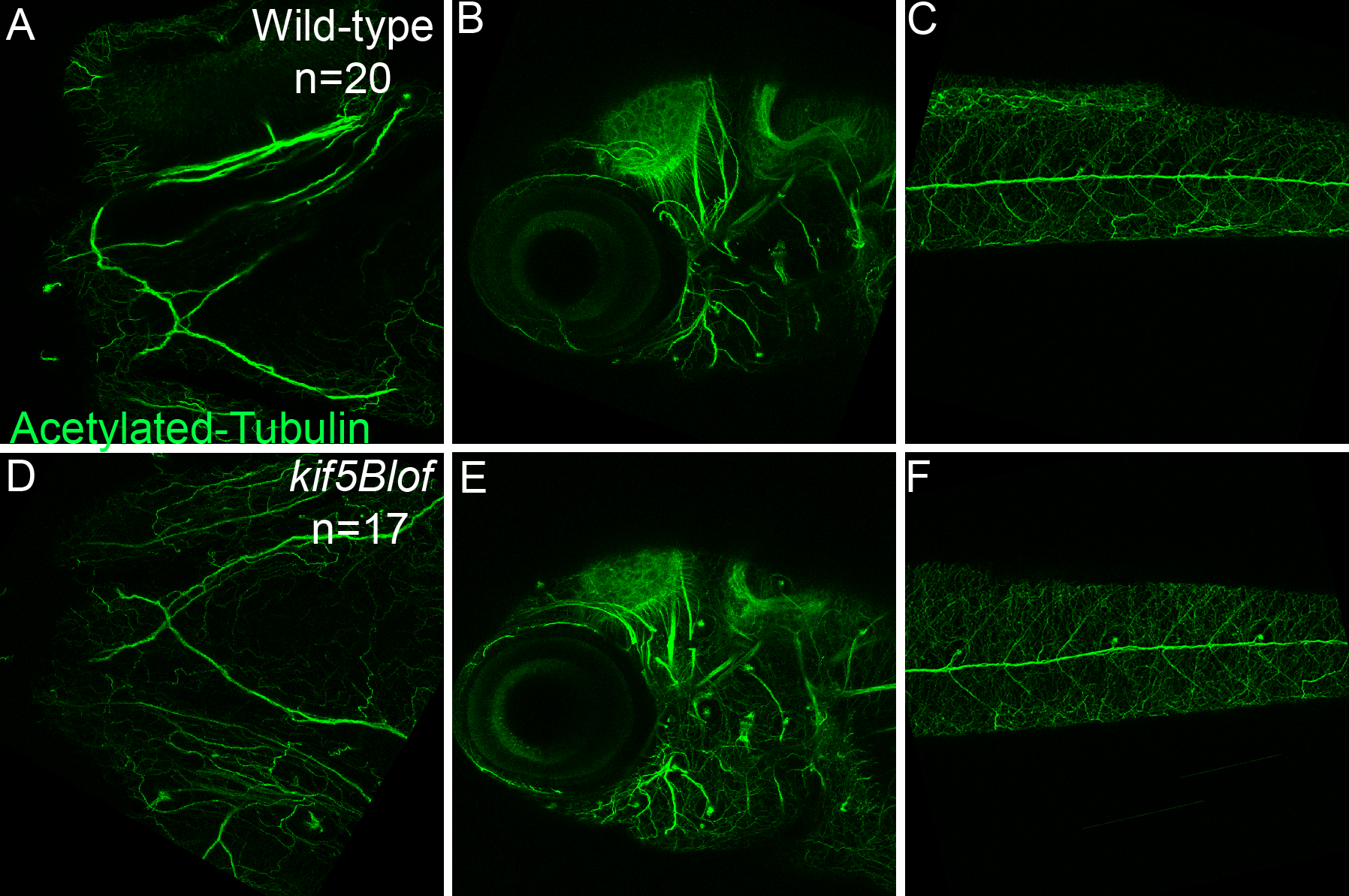

Supplement: S2 Fig — Ventral (A, D) or lateral (B, E) view of the head and lateral view of the tail (C, F) of Acetylated tubulin staining in Wt (A-C) and kif5Blof mutants (D-F). (TIF) [file pgen.1006918.s002.tif]

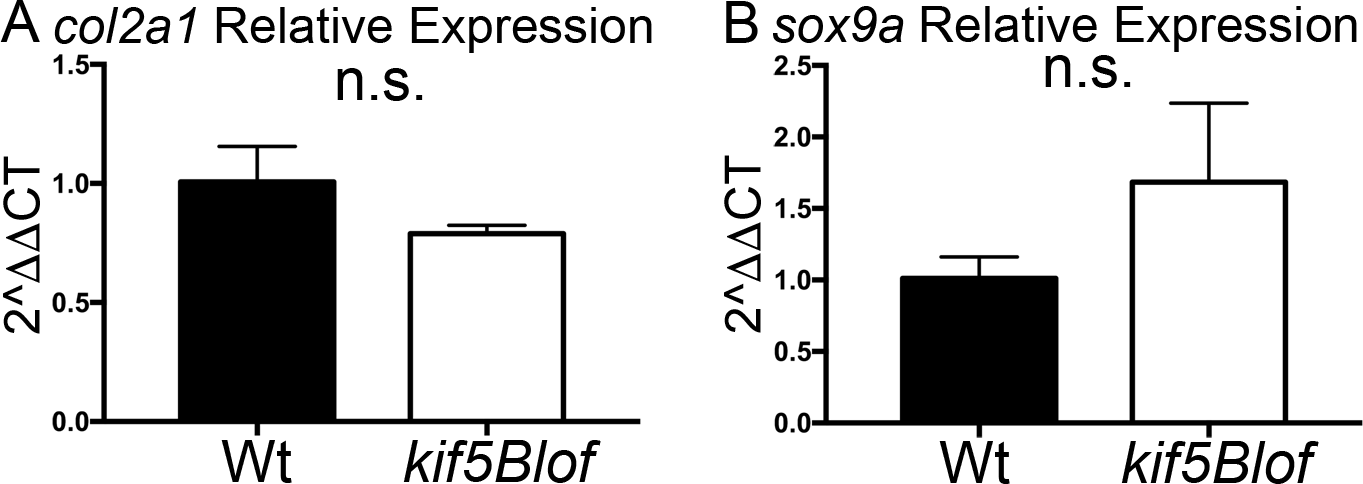

Supplement: S3 Fig — qRT-PCR for col2a1 (A) and sox9a(B) showed no differences between Wt and Kif5B mutants. n.s. No significant after Student T test. Three different batches of embryos were included in each group. (TIF) [file pgen.1006918.s003.tif]

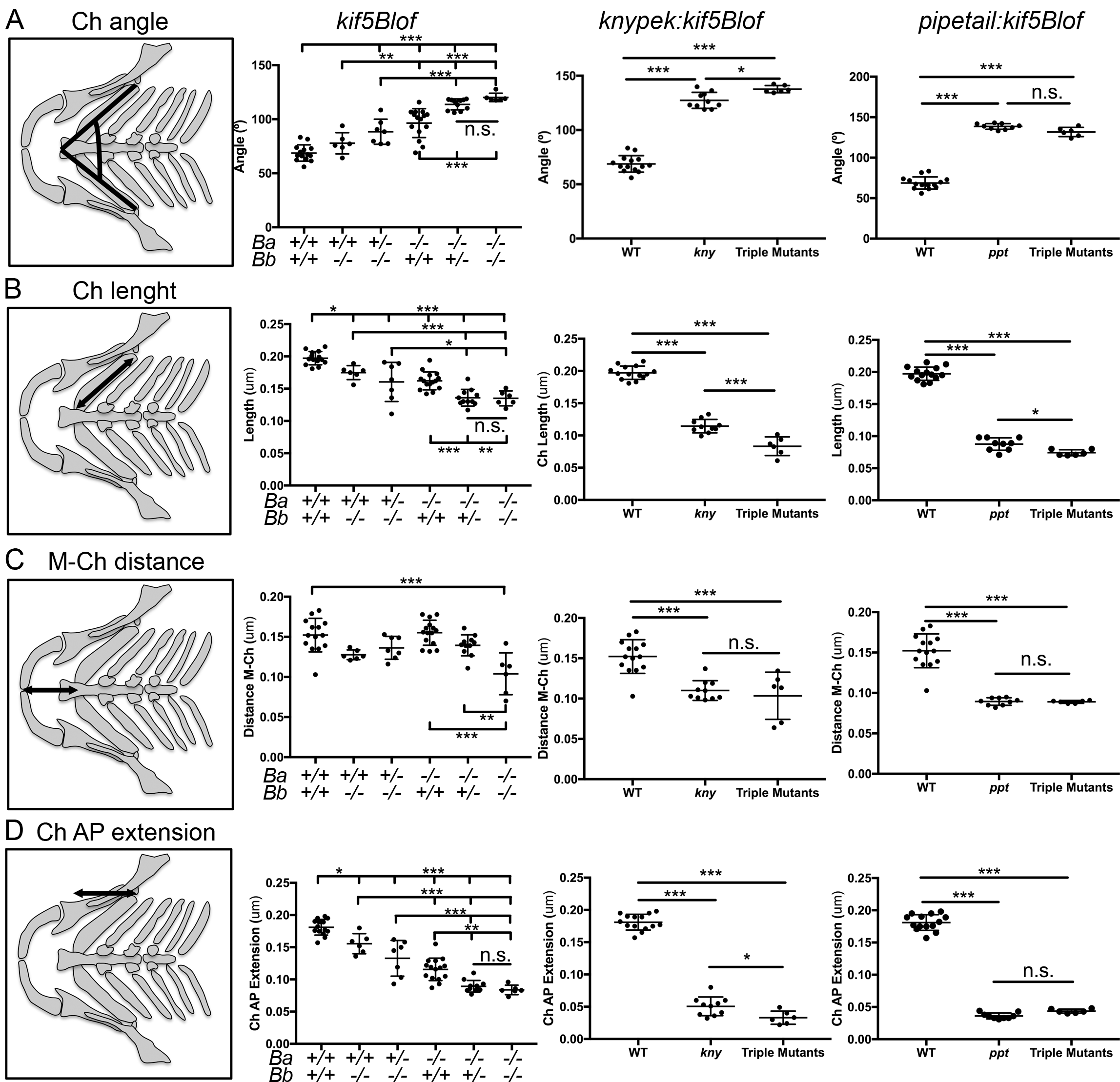

Supplement: S4 Fig — Quantification of the Ch angle (A), Ch length (B), the distance between Meckel´s and ceratohyal (C), and the extension of the ceratohyal cartilage along the antero-posterior axis. In kif5Blof mutants (second column), the angle was wider, the length was shorter, the M-Ch distance was shorter and the AP extension was reduced. In knypek mutants (third column) the angle was wider, the length and the AP extension were shorter, and these conditions were aggrevated in the triple mutants. In pipetail mutants (fourth column) the angle was wider, the length and the AP extension were shorter, and the ceratohyal was shorter in triple mutants. One-way ANOVA * p<0.05, ** p<0.01, *** p<0.001. AP: antero-posterior axis; Ch: ceratohyal; M: Meckel´s. (TIF) [file pgen.1006918.s004.tif]

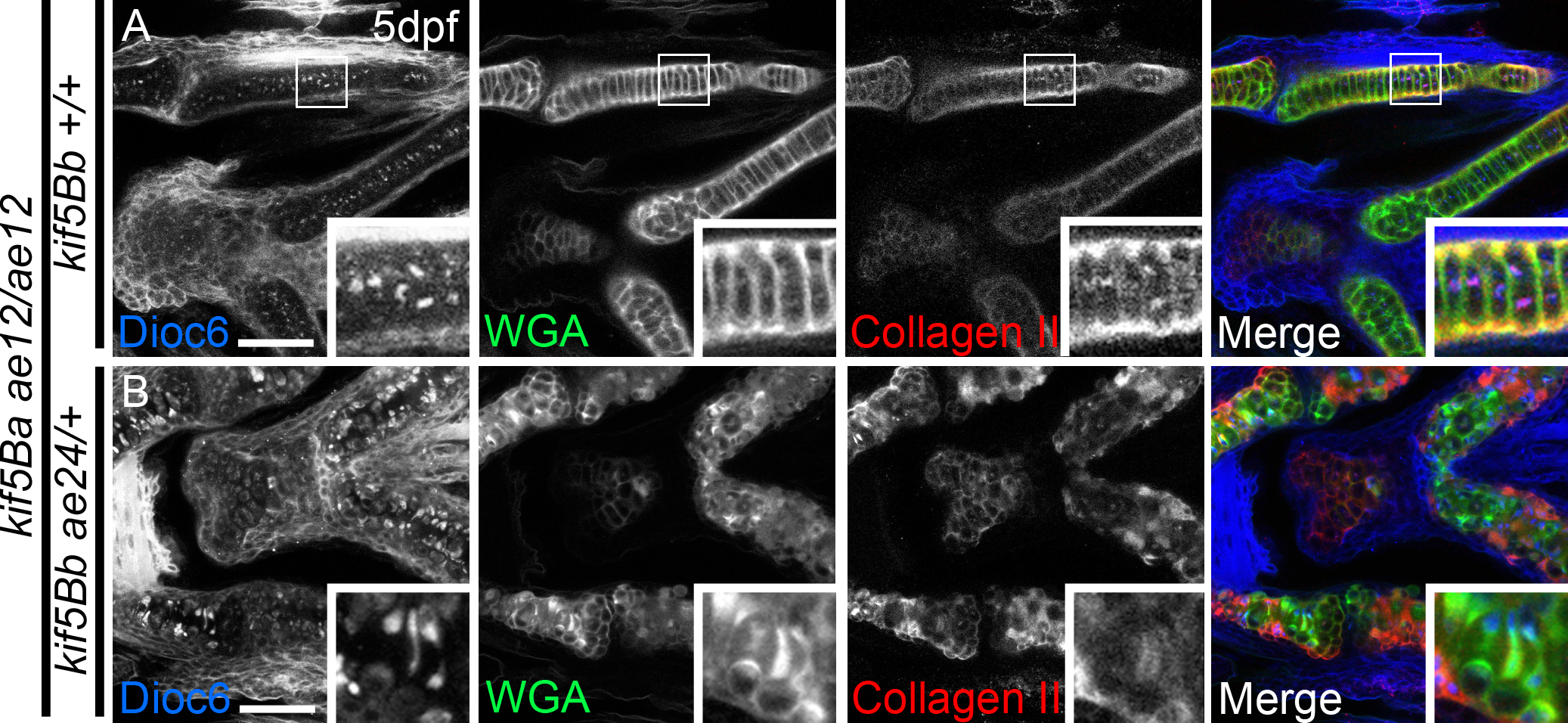

Supplement: S5 Fig — Some kif5B single mutants (kif5Baae12/ae12Kif5Bb+/+) resembled Wt in terms of membrane distribution and secretion (A). kif5B compound mutant (kif5Baae12/ae12Kif5Bbae24/+) phenotypes were fully penetrant and indistinguishable from double mutants. (TIF) [file pgen.1006918.s005.tif]

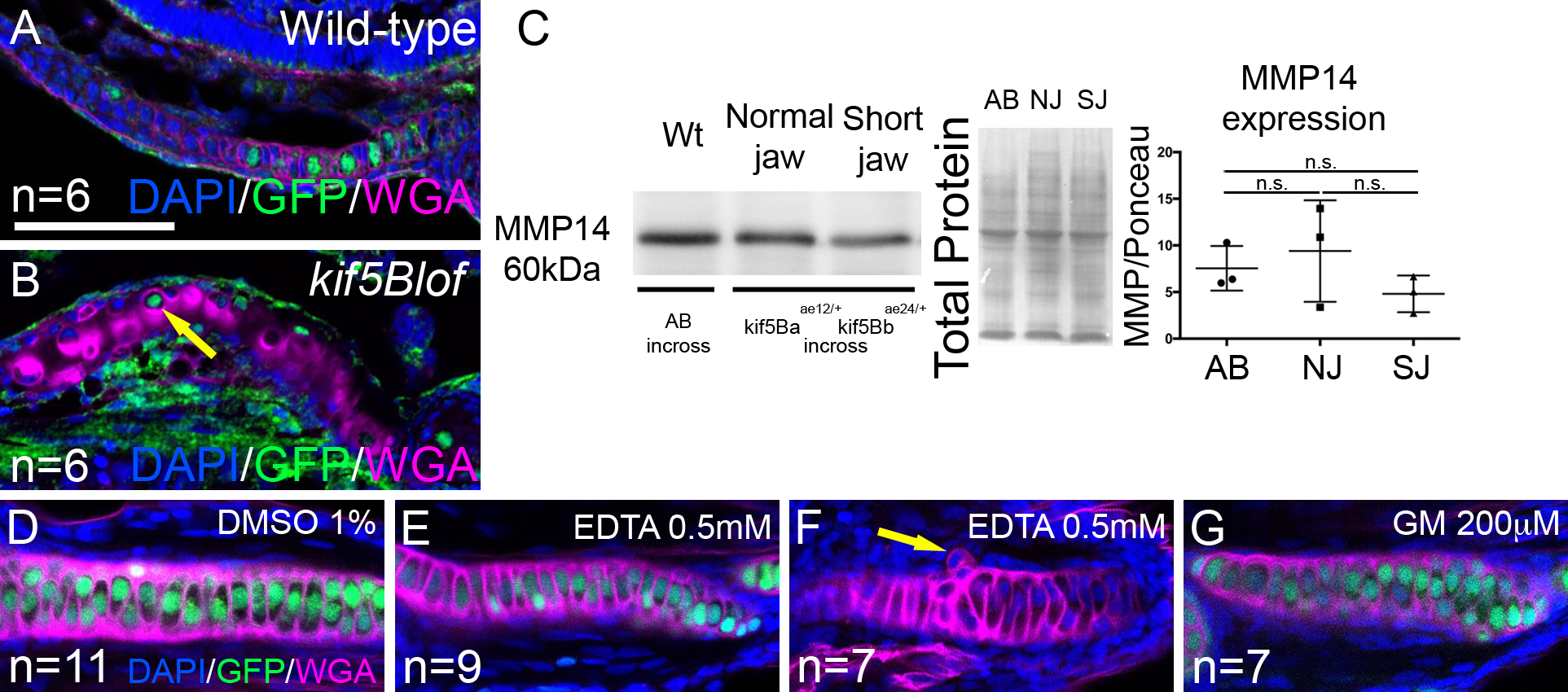

Supplement: S6 Fig — Chondrocytes and perichondrium are positive for MMP14 in Wt (A) and kif5Blof (B). Western blot reveals no significant changes [90] in MMP14 levels (C). Scale bar: 1 μm. D-G) Wt embryos show no kif5Blof-like phenotypes when exposed to broad-spectrum metalloproteinase inhibitor (E, F) or the MMP inhibitor GM6001 (G), although some cells were extruded from the cartilage at high concentrations of EDTA (arrow in F). (TIF) [file pgen.1006918.s006.tif]
